# Genetically Encoded SpyTag Enables Modular AAV Retargeting via SpyCatcher-Fused Ligands for Targeted Gene Delivery

**DOI:** 10.1021/acssynbio.5c00565

**Published:** 2025-12-22

**Authors:** Anja Armbruster, Maximilian Hörner, Hanna J. Wagner, Claudia Fink-Straube, Wilfried Weber

**Affiliations:** † INM − Leibniz Institute for New Materials, Saarbrücken 66123, Germany; ‡ Signalling Research Centres BIOSS and CIBSS, 98898 University of Freiburg, Freiburg i. Br. 79104, Germany; § Faculty of Biology, University of Freiburg, Freiburg i. Br. 79104, Germany; ∥ Department of Materials Science and Engineering, Saarland University, Saarbrücken 66123, Germany

**Keywords:** AAV capsid engineering, AAV retargeting, DARPins, modular gene delivery, SpyTag/SpyCatcher, suicide gene therapy

## Abstract

Recombinant adeno-associated viral (rAAV) vectors are a leading platform for *in vivo* gene therapy, valued for their excellent safety, broad serotype diversity, and scalable production. Targeted delivery through capsid display of ligands holds great promise, yet current retargeting strategies often rely on extensive capsid re-engineering and restrict the use of ligands incompatible with intracellular expression systems. Here, we present a modular AAV retargeting platform that, for the first time, employs the SpyTag/SpyCatcher system via genetic integration into the AAV2 capsid. SpyTag is a small peptide that forms a covalent, irreversible bond with its protein partner, SpyCatcher, allowing site-specific ligand coupling under physiological conditions. Inserting SpyTag into surface-exposed capsid sites enabled postassembly functionalization of AAVs with SpyCatcher-fused targeting proteins. As proof of concept, we used SpyCatcher fusions with designed ankyrin repeat proteins (DARPins) specific for EGFR, EpCAM, and HER2. This conferred highly specific transduction of corresponding cancer cell lines with minimal off-target activity. Therapeutic potential was demonstrated by delivering a suicide gene, inducing selective cancer cell killing upon prodrug administration. This “one-fits-all” platform allows rapid and flexible retargeting without significantly altering the underlying vectors genome or production process. It supports the incorporation of large or complex ligands not amenable to genetic fusion and facilitates high-throughput preclinical evaluation strategies. By uniting capsid engineering with modular ligand display, our approach provides a scalable and versatile framework for precision gene delivery, broadening the applicability of rAAV in both therapeutic and discovery settings.

## Graphical abstract

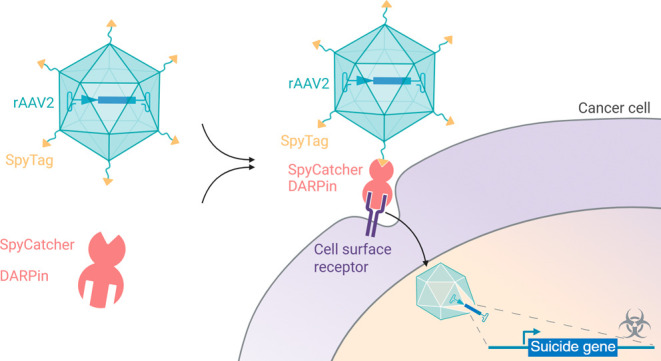

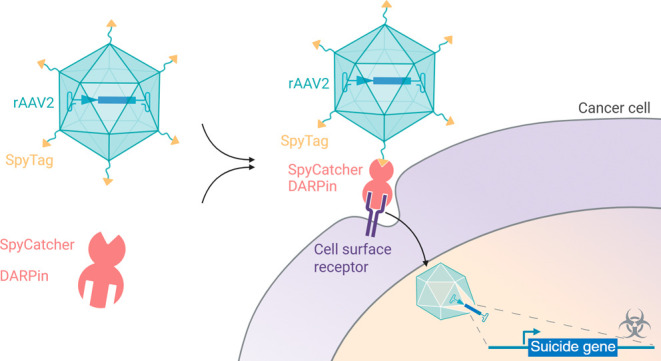

## Introduction

The 21st century has seen rapid advancements in gene therapeutics. Since the approval of Glybera, the first viral gene therapy in the US and Europe, at least 18 viral gene therapies have been marketed to treat cancer, rare monogenic diseases, hematological and neurological disorders, and more. These therapies leverage viruses’ natural capacity to deliver genetic material into target cells, enabling treatment at the genetic level.^1^ Among viral vectors, adeno-associated viral (AAV) vectors have emerged as the leading platform for *in vivo* gene therapy, as demonstrated by seven approved gene therapies, including Zolgensma, Luxturna, and Beqvez.^2,3^

AAVs popularity stems from several key features, including its limited ability to integrate into the hosts genome,^4,5^ their low immunogenicity and lack of pathogenicity^6^ as well as the availability of serotypes with different tissue specificities.^7^ Wild-type AAV (wtAAV) has a 4.7 kb single-stranded DNA genome flanked by inverted terminal repeats (ITRs) that function as packaging signals. The genome encodes genes for replication (*rep*) and structural capsid proteins (*cap*), including the proteins VP1, VP2, and VP3, which assemble into a 60-mer capsid.^8,9^ While wtAAV infection can result in latent genome integration,^4,5^ recombinant AAV (rAAV) vectors used in gene therapy remain episomal in transduced cells, minimizing the risk of insertional mutagenesis.^10^

Beyond natural AAV serotypes, engineered rAAV capsids are being developed for clinical applications by using genetic or chemical modifications and peptide insertions to achieve altered tissue specificities or capsid shielding.^11–13^ AAV capsids can be functionalized by two primary strategies. Small peptides or protein domains are commonly inserted into exposed surface loops. In AAV2, position 587 in variable region VIII is the most utilized insertion site, enabling targeting of specific cell types by display of targeting ligands.^14–16^ Insertions at this site also ablate natural tropism by disrupting primary receptor (heparan sulfate) binding.^17,18^ Larger inserts, such as nanobodies for targeting cell-surface receptors, can be accommodated in variable region IV.^19^ Alternatively, larger peptides or proteins can be fused to the N-terminus of VP2 and exposed through a pore in the viral capsid, allowing the display of affibodies, designed ankyrin repeat proteins (DARPins), or single-chain variable fragments (scFvs) for precise targeting.^20,21^

While genetic fusion strategies have enabled progress in targeted AAV gene delivery, they face critical limitations. Each new target requires vector redesign, which is labor-intensive, results in a high variability of vectors, and necessitates extensive characterization. Additionally, AAV capsids tolerate only limited insertions: surface-exposed loops and the VP2 N-terminus typically accommodate only peptides or small proteins, while larger proteins often destabilize the capsid or interfere with assembly. Furthermore, targeting ligands are restricted to proteins and molecules stable in the reducing environment of the host cell nucleus during vector production, excluding many antibody fragments and native ligands. Alternative strategies like bispecific antibodies^22^ or chemical coupling strategies like biotinylation^23^ often lack the covalent binding stability needed for *in vivo* applications or require additional chemical^24^ or enzymatic steps (e.g., BirA-mediated biotinylation^25^).

The SpyTag/SpyCatcher system, derived from a split fragment of the *Streptococcus pyogenes* fibronectin-binding protein FbaB,^26^ provides a compelling solution. This system enables site-specific covalent coupling of a peptide tag (SpyTag) with its protein partner (SpyCatcher) under physiological conditions without the need for cofactors or chemical catalysis. Previous studies have leveraged this system to equip viral particles postassembly. For example, Kasaraneni *et al.* ^27^ inserted a SpyTag into the envelope of Sindbis-pseudotyped lentiviral vectors and redirected them to HER2+ cells using SpyCatcher fused to HER2-specific DARPins or Fab' fragments. Similarly, Kadkhodazadeh *et al.* ^28^ used SpyTag insertion into the HI loop of the adenovirus type-5 (Ad5) fiber knob to enable modular targeting via SpyCatcher-nanobody conjugates. More recently, Zhang *et al.* ^29^ equipped AAV2 with SpyTags by inserting an unnatural amino acid into VP3 and coupled the vector to SpyCatcher-fused nanobodies via a SpyTag-click-chemistry adapter, enabling targeted gene delivery *in vitro* and *in vivo*.

In this study, we present a genetically integrated SpyTag displayed directly in the AAV capsid, enabling covalent and modular redirection of rAAV2 via postassembly coupling to SpyCatcher-ligand fusions. This approach unites the advantages of genetic engineering and modular postproduction functionalization: a single, universal SpyTag-AAV vector that can be redirected to diverse cell types simply by exchanging the SpyCatcher-fused targeting ligand.

We demonstrate the flexibility of this platform by equipping SpyTag-AAVs with DARPins targeting EGFR, EpCAM, and HER2, which successfully redirected the vector to the corresponding cancer cell lines. Furthermore, we validated the therapeutic functionality through the delivery of a suicide gene and subsequent prodrug-induced cell death. By decoupling targeting from vector production, our platform streamlines the rapid screening and preclinical evaluation of new AAV-based therapeutics. The ability to test diverse ligands without re-engineering the capsid accelerates discovery and facilitates the development of receptor-specific gene delivery strategies.

## Results

### Design of a Modular AAV-Based Transduction Platform

Our transduction platform comprises an engineered recombinant AAV2 (rAAV2) and an exchangeable targeting ligand that mediates the selective binding of a cellular receptor and thereby targeted transduction of cells (Figure 1A). The AAV vector carries a mutation in its heparan sulfate proteoglycan (HSPG) binding motif, which disables primary receptor binding,^17,18^ and displays a genetically inserted SpyTag peptide^26^ on its capsid. The targeting ligand comprises a fusion protein of the SpyTag-binding partner SpyCatcher^26,30^ and a designed ankyrin repeat protein (DARPin) specific for a cell surface receptor.^31^ The SpyCatcher forms an amide bond with the SpyTag, thereby equipping the AAV with the DARPin. The DARPin mediates specific interactions with a cell surface receptor, resulting in selective transduction of the target cell.

**Figure 1:**
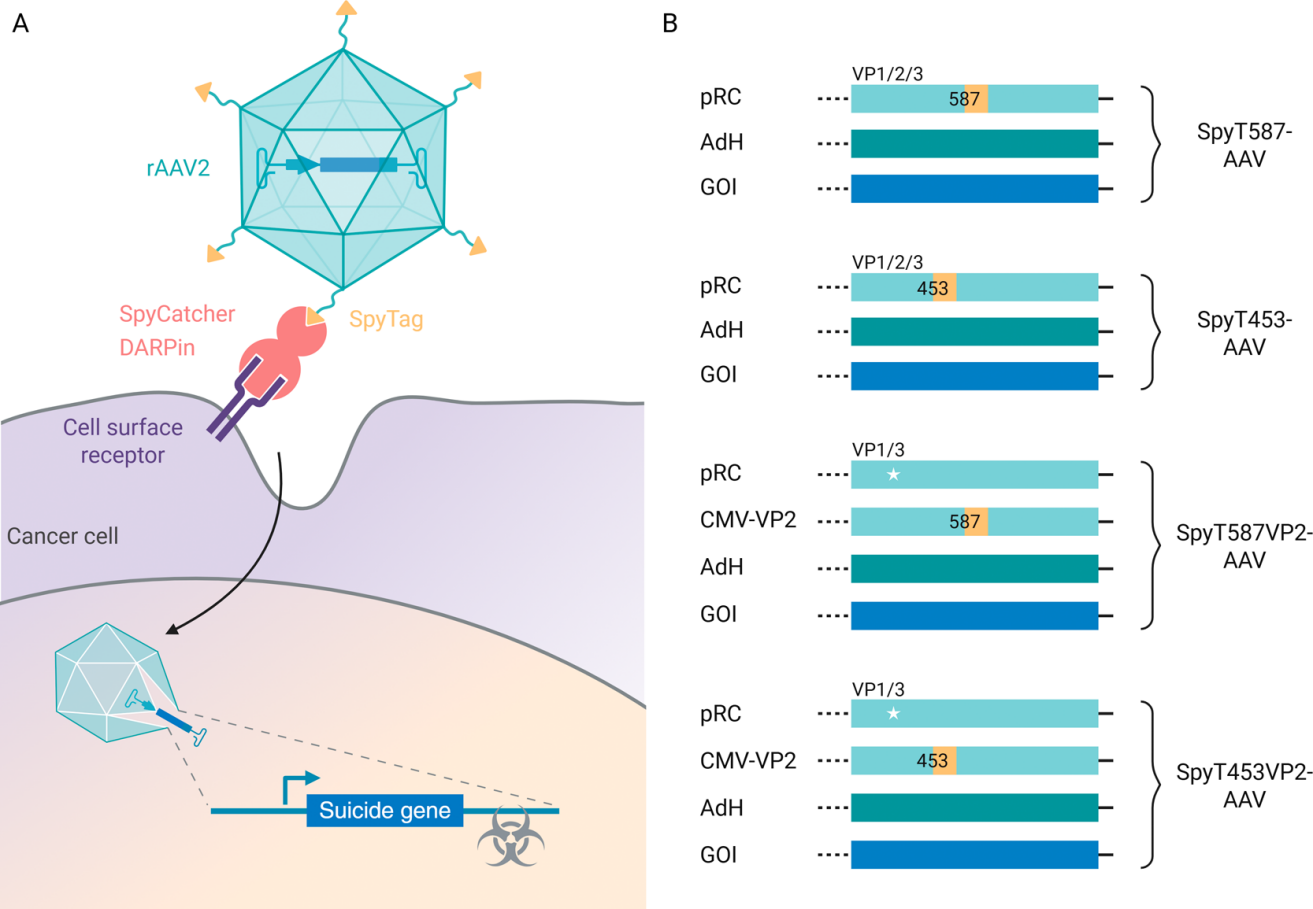
Design of a modular AAV-based transduction platform for cancer cell therapy. (A) Schematic illustration of the modular AAV retargeting platform designed for cancer cell transduction. The rAAV2 capsid displays a genetically inserted SpyTag, which binds to a SpyCatcher-DARPin fusion protein. The DARPin binds to cancer cell surface receptors, enabling the delivery of a suicide gene that triggers cell death upon prodrug administration. (B) Overview of SpyTag-AAV constructs used for AAV production. The SpyTag001 peptide (AHIVMVDAYKPTK) (yellow) was inserted at positions 587 or 453 of the viral capsid, either in all three viral capsid proteins (VP1, VP2, and VP3) or exclusively into VP2 with a pRC plasmid harboring a mutated VP2 start codon (white star) supplied *in trans*. Abbreviations: AdH, adenovirus helper plasmid; DARPin, designed ankyrin repeat protein; GOI, gene of interest; pRC, rep–cap plasmid; rAAV, recombinant adeno-associated virus; and SpyT, SpyTag.

### Implementation and Characterization of the Transduction Platform

To implement and characterize the modular AAV-transduction platform, we selected DARPin E_01, a designed ankyrin repeat protein (DARPin) specific for the epidermal growth factor receptor (EGFR), which is commonly expressed on tumor cells.^32,33^ DARPin E_01 has previously been utilized to retarget viral vectors, including AAVs, to EGFR-expressing cells.^34–36^ We produced the SpyCatcher-DARPin_EGFR_ fusion protein (referred to hereafter as SpyC-DARPin_EGFR_) in *Escherichia coli*, purified it using immobilized metal ion affinity chromatography (IMAC), and confirmed its integrity via SDS-PAGE and Coomassie staining (Figure S1).

For the genetic integration of the SpyTag into the rAAV2 capsid, we used two sites: positions 587 in the variable region VIII^14^ and 453 within the variable region IV.^37^ Both positions are located in surface-exposed loops of the AAV capsid and have previously demonstrated tolerance for peptide insertions for rational targeting of cells.^14–16,37,38^ We inserted SpyTag at both positions in two configurations: (i) into all three viral capsid proteins (VP1, VP2, and VP3) using plasmids pMH321 and pHJW414 (see Tables S7 and S8) and (ii) exclusively into VP2 using plasmids pHJW341 and pHJW351. For the latter, VP2 expression was driven by a CMV promoter *in trans* to a rep–cap plasmid harboring a mutated VP2 start codon (plasmid pHJW162), preventing the production of unmodified VP2. To ablate the natural tropism of the AAV vectors and ensure transduction only in the presence of the DARPin, we introduced R585A and R588A mutations into the cap gene.^18^ ,^39^ This process yielded four distinct AAV variants: SpyT587-AAV, SpyT453-AAV, SpyT587VP2-AAV, and SpyT453VP2-AAV (Figure 1B and Table S7).

SpyTag-AAVs carrying the fluorescent reporter gene mScarlet (plasmid CMV-mScarlet) were produced using a helper-free packaging system in HEK-293T cells and precipitated from cell culture supernatant using polyethylene glycol (PEG) (see Methods). The genomic titers of SpyTag-AAV2 variants were comparable to those of wild-type AAV2 (wtAAV2). Typical titers of wtAAV2 and SpyTag-AAV2 productions are listed in Table S6. Western blot analysis against the viral capsid proteins VP1, VP2, and VP3 confirmed the presence of the viral capsid proteins in all AAV preparations (Figure 2).

**Figure 2:**
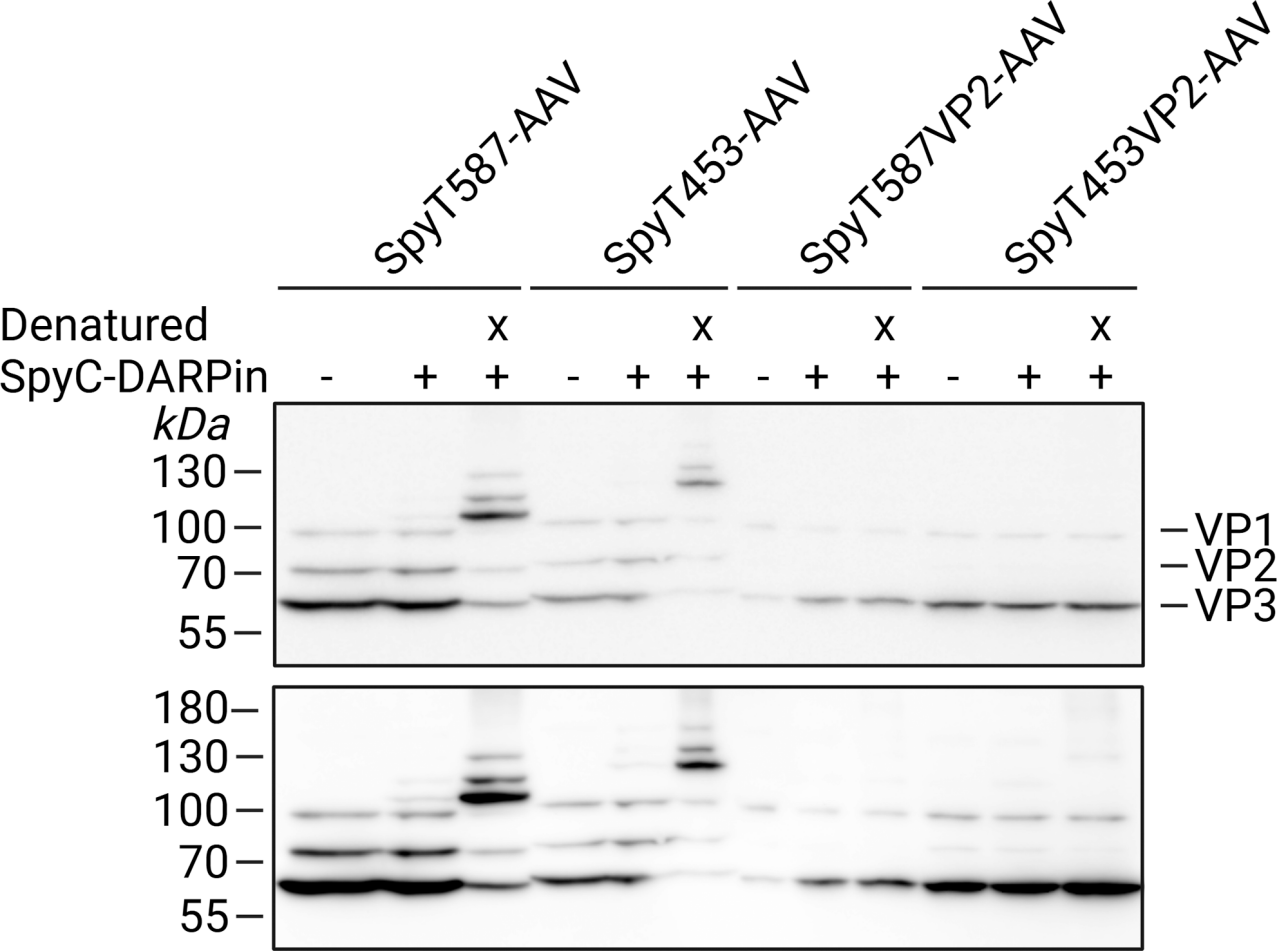
Evaluation of capsid composition and SpyCatcher-DARPin_EGFR_ coupling of SpyTag-AAVs. Western blot analysis of PEG-precipitated viral capsid proteins VP1, VP2, and VP3 of SpyT587-AAV, SpyT453-AAV, SpyT587VP2-AAV, and SpyT453VP2-AAV from cell culture supernatant. “+” indicates incubation with SpyCatcher-DARPin_EGFR_, “x” denotes AAV denaturation by boiling at 98 °C for 10 min before SpyC-DARPin coupling. The bottom panel shows an overexposed version of the membrane presented in the upper panel, revealing weaker signals. SpyT-AAV and SpyC-DARPin_EGFR_ concentrations are listed in Table S1.

To assess SpyC-DARPin coupling, precipitated SpyT-AAVs were incubated with SpyC-DARPin either directly or following heat denaturation (98 °C, 10 min), which served to expose any inaccessible SpyTags. Successful coupling was indicated by a molecular weight shift in capsid proteins on the Western blot due to the covalent attachment of the SpyC-DARPin.

Both SpyT587-AAV and SpyT453-AAV displayed clear VP1, VP2, and VP3 signals (Figure 2, upper panel), while SpyT587VP2-AAV and SpyT453VP2-AAV exhibited only a faint VP2 band (Figure 2, lower panel). These two constructs rely on *trans*-complementation of SpyTag-modified VP2, which can disturb the canonical ∼1:1:10 VP1/VP2/VP3 stoichiometry. Consistent with this, densitometric analysis of VP bands (Table S2) revealed differences in VP subunit incorporation between the four vectors, in particular, reduced VP2 content in the VP2-SpyTag constructs. Furthermore, SpyT587-AAV and SpyT453-AAV displayed additional higher molecular weight bands after SpyC-DARPin_EGFR_ incubation both in their native, albeit weak, and their denatured state, while SpyT587VP2-AAV and SpyT453VP2-AAV only displayed weak additional bands after denaturation. Attempts to increase SpyTag accessibility (weak denaturants, mildly acidic buffer, limited heat-shock) did not enhance ligand display and instead impaired AAV functionality, suggesting that partial capsid destabilization does not improve coupling efficiency under the tested conditions.

The low coupling efficiency in the native state with notable coupling to denatured capsid proteins confirms that SpyTags were correctly integrated but likely buried within the intact capsid structure. This suggests that only a small subset of viral proteins exposed accessible SpyTags on the capsid surface.

To assess whether coupling SpyC-DARPin_EGFR_ to the SpyTag-AAVs enabled specific retargeting, we evaluated the vectors’ ability to transduce A-431 cells, which overexpress EGFR. To this aim, we assessed transduction efficiency at different SpyT-AAV doses and at different SpyC-DARPin protein concentrations. Serial dilutions of the vectors were preincubated with SpyC-DARPin_EGFR_ in cell culture medium before being added to the cells (Figure 3). Flow cytometry analysis revealed that the coupling of SpyC-DARPin_EGFR_ enabled targeting of SpyT-AAVs to target cells. The dilution series of SpyT-AAVs showed a vector-concentration dependent increase in mScarlet-positive cells, indicating successful transduction across all four capsid variants tested. Interestingly, despite the absence of detectable SpyC-DARPin_EGFR_ coupling in Western blot analysis, SpyT587VP2-AAV and SpyT453VP2-AAV achieved transduction efficiencies comparable to those of SpyT587-AAV and SpyT453-AAV at the highest tested multiplicity of infection (MOI). Notably, SpyT453-AAV maintained robust transduction even at MOIs as low as 190, underscoring its efficiency. Specificity was confirmed by the minimal off-target transduction observed in AAVs incubated without SpyC-DARPin_EGFR_ (<6.2% for SpyT453-AAV and <1.2% for all others at MOI 1.3 × 10^4^, Figure 3), highlighting that the retargeting mechanism depended on the SpyC-DARPin_EGFR_ interaction. To assess whether the increased transduction observed in the presence of SpyC-DARPin was dependent on covalent coupling to SpyT-AAV, we performed two complementary controls (Figure S2). First, A-431 cells were transduced with wtAAV2 in the presence or absence of SpyC-DARPin_EGFR_ (Figure S2A). wtAAV2 transduction remained essentially unchanged across conditions, indicating that DARPin alone did not enhance infectivity in the absence of a SpyTag on the capsid. Second, we transduced CHO-K1 cells, which do not express human EGFR, with SpyT453-AAV equipped with different SpyCatcher-DARPin_EGFR_ variants (Figure S2B). In this setting, transduction efficiencies remained within the same low range as uncoupled SpyT453-AAV. Together, these results showed that the DARPin ligand did not increase infectivity on its own and that efficient redirection required the covalent linkage of the SpyC-DARPin to SpyTag-AAV.

**Figure 3:**
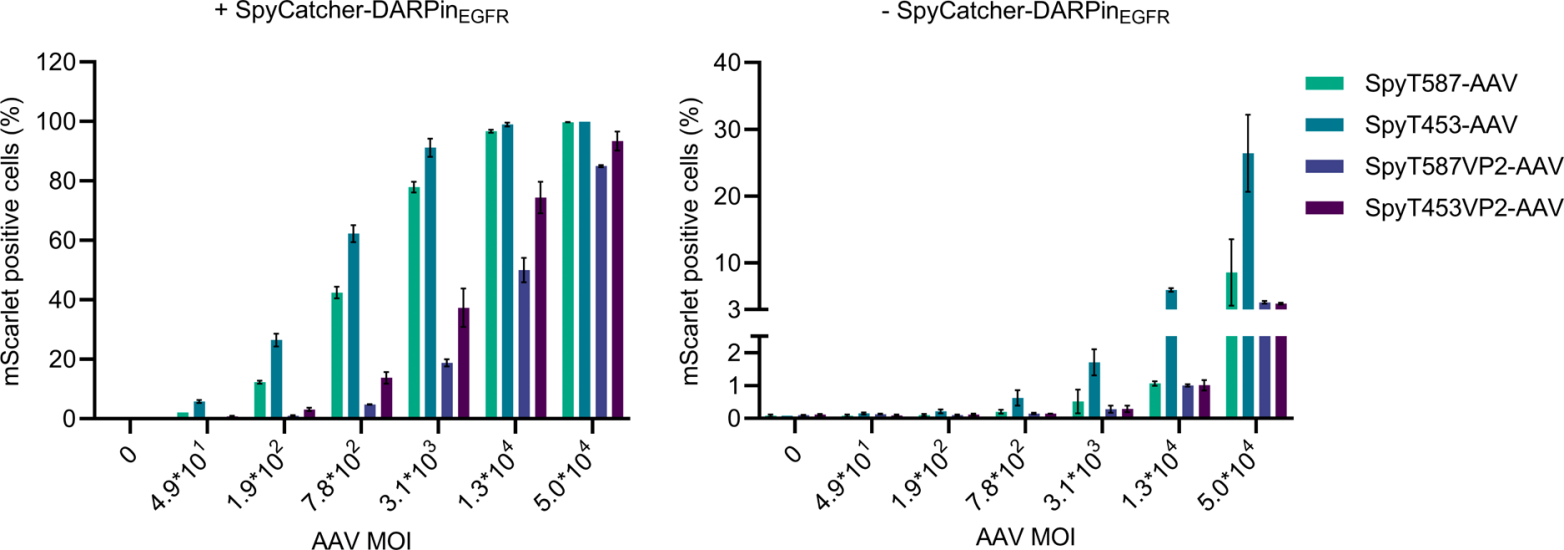
SpyCatcher-DARPin_EGFR_ specific targeting of EGFR-overexpressing A-431 cells. Indicated SpyTag-AAV variants were evaluated for their transduction of EGFR-overexpressing A-431 cells with or without SpyCatcher-DARPin_EGFR_. AAVs were serially diluted (starting from 2.0 × 10^9^ vg/mL, then diluted serially 1:4) and each dilution was incubated with 100 nM of SpyC-DARPin_EGFR_ for 1 h at 37 °C. Transduction mix was then added to A-431 cells and transduction efficiency was analyzed after 48–72 h by quantifying mScarlet positive cells by flow cytometry. Bars represent means ± SD of *n* = 3 biological replicates.

Although integration site 587 has been widely utilized for peptide insertions into the AAV capsid, position 453 has not yet gained the same popularity.^9,40^ Given that SpyT453-AAV demonstrated the highest transduction efficiency of all four AAVs and retained efficiency even at low MOIs, we selected SpyT453-AAV for subsequent experiments to further explore its potential in targeted gene delivery.

We next sought to identify the optimal concentration and coupling conditions for SpyC-DARPin_EGFR_ to maximize the SpyT453-AAV-mediated transduction. Serial dilutions of SpyC-DARPin_EGFR_ were incubated with SpyT453-AAV for 1 h at 37 °C or overnight at room temperature (Figure 4A). Transduction of A-431 cells was efficient across a broad range of SpyC-DARPin_EGFR_ concentrations (1 μM to 1 nM) with nonsignificant differences between temperatures and incubation times; therefore, the 37 °C and 1 h condition was used for all subsequent experiments. Peak transduction efficiency was observed at 10 nM SpyC-DARPin. At higher concentrations, transduction declined, most likely due to receptor blockage by excess free SpyC-DARPin_EGFR_ rather than steric overload of the capsid, which would be consistent with the low fraction of functionally accessible SpyTags observed in Figure 2. Below 1 nM, transduction also dropped markedly, suggesting inefficient SpyTag-SpyCatcher coupling under these conditions.

**Figure 4:**
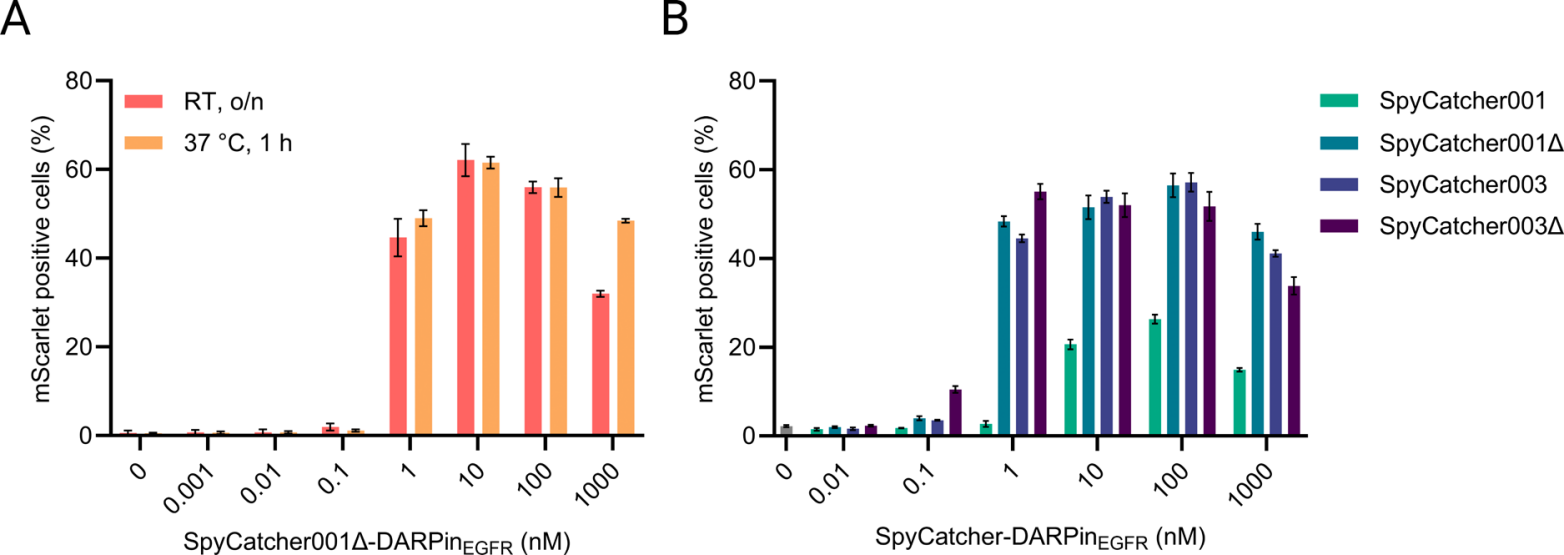
Influence of SpyCatcher coupling conditions and variants on SpyTag-AAV transduction efficiency. (A) Titration of the SpyCatcher-DARPin_EGFR_ concentration. SpyT453-AAV (3.1 × 10^7^ vg/mL, MOI: 7.8 × 10^2^) was preincubated with serial dilutions of SpyC-DARPin_EGFR_ for 1 h at 37 °C or overnight (o/n) at room temperature prior to the transduction of A-431 cells. (B) Evaluation of SpyCatcher variants on SpyTag-AAV transduction efficiency. SpyT453-AAV (1.0 × 10^8^ vg/mL, MOI: 2.5 × 10^3^) was preincubated with serial dilutions of various SpyCatcher-DARPin_EGFR_ fusions for 1 h at 37 °C. Transduction efficiency was measured as mScarlet positive A-431 cells by flow cytometry. Bars represent means ± SD of *n* = 3 biological replicates.

To assess whether excess unbound ligand could be removed, a step that would be required for translational use, we dialyzed the coupling reactions against PBS using a membrane with a 1000 kDa molecular weight cutoff (MWCO). This removed free SpyC-DARPin_EGFR_ while retaining SpyC-DARPin_EGFR_-coupled SpyT-AAV as confirmed by Western blot (Figure S3A,B), and the recovered complexes remained functional in A-431 cells (Figure S3C). Transduction efficiency after dialysis was reduced relative to that of the crude coupling mix due to sample dilution during dialysis. Because excess SpyCatcher did not influence transduction at the concentrations used in this study and dialysis reduced particle recovery, ligand removal was not routinely performed in subsequent experiments.

Thus far, we had employed the truncated SpyCatcher001ΔN3ΔC2 ^30^ (referred to hereafter as SpyCatcher001Δ) for coupling. SpyCatcher001Δ was chosen for its reported reduced interaction with cell surface receptors compared to SpyCatcher001.^27^ To explore whether alternative SpyCatcher variants could enhance transduction, we generated and tested DARPin_EGFR_ fusions with SpyCatcher001,^26^ SpyCatcher003, which harbors a stabilized loop and increased surface polarity for enhanced reaction compared to the first generation,^41^ and our newly engineered variant, SpyCatcher003Δ, designed through sequence comparisons of SpyCatcher001 and SpyCatcher001Δ (Figures S4 and S5). All variants enabled efficient A-431 cell transduction, but none considerably outperformed Spycatcher001Δ, except for SpyCatcher003Δ in one instance, showing a modest advantage at 1 nM (Figure 4B). Since the SpyC-DARPin_EGFR_ construct did not critically influence overall transduction efficiency, we chose to continue using Spycatcher001Δ for subsequent experiments.

### Modular Redirection of AAV to Different Cancer Cells

To demonstrate that our AAV platform enables modular and versatile retargeting to different cell lines, we replaced the EGFR-specific DARPin E_01 with DARPin Ec1 ^42^ and DARPin 9_29,^32^ which specifically target EpCAM and Her2/ErbB2, respectively (Figure S6). As a negative control, we included a CD4-specific DARPin D55.2 ^43^ (Figure S6), a receptor absent from our tested cell lines.

SpyT453-AAV equipped with DARPin E_01 selectively transduced A-431 and SK-OV-3 cells, both of which express EGFR^44,45^ (Figure 5). DARPin Ec1 enabled specific redirection to EpCAM-expressing MDA-MB-453 cells^46,47^ and, to a lesser extent, to A-431 cells, which express low levels of EpCAM.^48^ Finally, DARPin 9_29 efficiently redirected AAVs to HER2-expressing SK-OV-3 cells^49,50^ as well as MDA-MB-453 cells, which are presumed to be HER2-positive.^51–54^ The differences in transduction efficiencies observed for individual DARPin-cell line combinations likely reflect the respective surface receptor expression levels in these cell lines and are consistent with receptor expression profiles reported in the literature. A very similar transduction pattern was observed in another study utilizing the same targeting ligands and cell lines but a different coupling mechanism to AAV altogether.^36^ SpyT453-AAV equipped with DARPin D55.2 transduced in the same low range as the ligand-free vector (Figure 5, AAV only).

**Figure 5:**
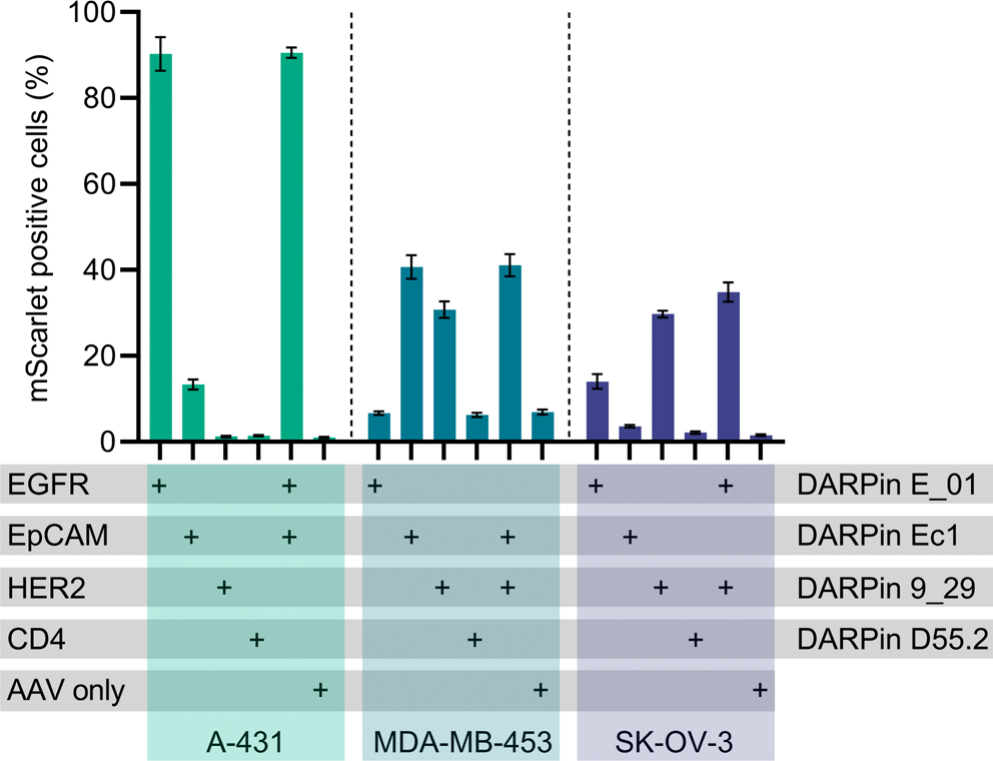
Modular retargeting of SpyTag-AAV to different cancer cell lines using surface marker-specific DARPins. SpyT453-AAV (1.4 × 10^8^ vg/mL, MOI: 3.5 × 10^3^) was preincubated with 100 nM of SpyC-DARPin fusions targeting EGFR (DARPin E_01), EpCAM (DARPin Ec1), Her2/ErbB2 (DARPin 9_29), CD4 (DARPin D55.2), or with a mix of two DARPins at 50 nM each. SpyT453-AAV without the targeting ligand served as the negative control (AAV only). A-431, MDA-MB-453 (EpCAM and HER2 expressing), and SK-OV-3 cells (Her2/ErbB2 expressing) were transduced. Transduction efficiency was determined by flow cytometry based on mScarlet expression. Bars represent means ± SD of *n* = 3 biological replicates.

To investigate whether targeting SpyT453-AAV with more than one ligand affected transduction, SpyC-DARPins were mixed pairwise at an equimolar concentration (50 nM + 50 nM) and coupled to SpyT453-AAV prior to transduction. We assessed three combinations (DARPins E_01 + Ec1, DARPins Ec1 + 9_29, and DARPins E_01 + 9_29) on A-431, MDA-MB-453, and SK-OV-3 cells. Across all cell types, the mixed-ligand conditions yielded transduction levels that remained within the same general range as that of the best transducing single DARPin for that cell line. Qualitatively, the values observed were close to a Bliss independence expectation *f*expected = 1–(1–*fA*)­(1–*fB*), i.e., compatible with two likely independent binding opportunities on the same capsid. Importantly, the combined conditions did not systematically exceed the Bliss expectation, indicating that we did not observe synergy beyond the independent contributions of the two ligands. Likewise, mixtures did not show systematic inhibition relative to the best transducing single DARPin. Given that all three cell lines express multiple tested receptors to varying and partly overlapping degrees, it was not possible to attribute relative contributions of each DARPin to a single receptor species. Nevertheless, these data demonstrated that AAV particles could be equipped with more than one targeting ligand simultaneously, and in this experimental setting, the resulting redirection could be approximated by the additive behavior of two independent recognition determinants.

Overall, SpyT453-AAVs functionalized with SpyC-DARPin fusion proteins efficiently transduced target-expressing cancer cells while maintaining minimal off-target transduction in nontarget expressing cell lines. Additionally, SpyTag/SpyCatcher allowed multiplexed surface functionalization of AAV and multiple targeting ligands could contribute to uptake without evidence of interference or synergy outside of simple additive behavior within the tested range. These results underscore the flexibility of the platform in retargeting AAV vectors to diverse cell types via modular DARPin substitutions.

### Targeted Killing of Cancer Cells Using a Suicide Gene Approach

As proof of concept for the modular retargeting, we applied our AAV system for virus-directed enzyme prodrug therapy (VDEPT, or suicide gene therapy), wherein an exogenous enzyme is delivered into, e.g., cancer cells, which then sensitizes the target cells to a nontoxic prodrug. Linamarase, an enzyme derived from cassava, has previously attracted attention for its anticancer properties and suicide gene potential.^55–57^ Linamarase catalyzes conversion of the cyanoglucoside linamarin into cytotoxic hydrogen cyanide (HCN).^58^ Cyanide induces cell death by blocking oxidative phosphorylation in the mitochondrial respiratory chain^59,60^ and kills surrounding cells through a bystander effect^61,62^ often desired in the treatment of tumor sites.

To leverage our modular platform for virus-directed enzyme prodrug therapy, we packaged the suicide gene linamarase into the SpyT453-AAV capsid. To this aim, the coding sequence for linamarase followed by a 2A peptide (T2A) was inserted upstream of the mScarlet reporter gene of plasmid CMV-mScarlet (resulting in plasmid pHJW427), allowing for coexpression of both genes. The resulting AAVs were characterized through Western blot analysis of the capsid proteins and by transduction efficiency assays in A-431 cells after coupling with SpyC-DARPin_EGFR_ (Figure S7).

A-431 cells were transduced with SpyC-DARPin_EGFR_-coupled SpyT453-AAVs carrying either linamarase-mScarlet fusion or mScarlet alone as the control before adding different concentrations of linamarin. Cell viability was evaluated using Zombie violet live/dead staining and flow cytometry (Figure 6). Additionally, hydrogen cyanide (HCN) production, a key indicator of linamarase activity, was quantified in the cell culture supernatant via gas chromatography mass spectrometry (GCMS) (Figure 6). While the percentage of live cells of mScarlet control transduced cells remained relatively constant in the presence of increasing linamarin concentrations, a continuous decrease was evident for linamarase-mScarlet transduced cells. A significantly decreased live cell number was seen for linamarin concentrations between 1000 and 2000 μg/mL linamarin. Proportionally, the amount of HCN produced by cells increased with increasing linamarin concentrations in the presence of linamarase only, reaching up to 241 μg/mL HCN. This equals a highly efficient average substrate conversion efficiency of approximately 98% (Table S5). These experiments confirmed successful gene delivery and enzymatic activity, providing a proof of concept for the use of SpyT453-AAVs in VDEPT applications.

**Figure 6:**
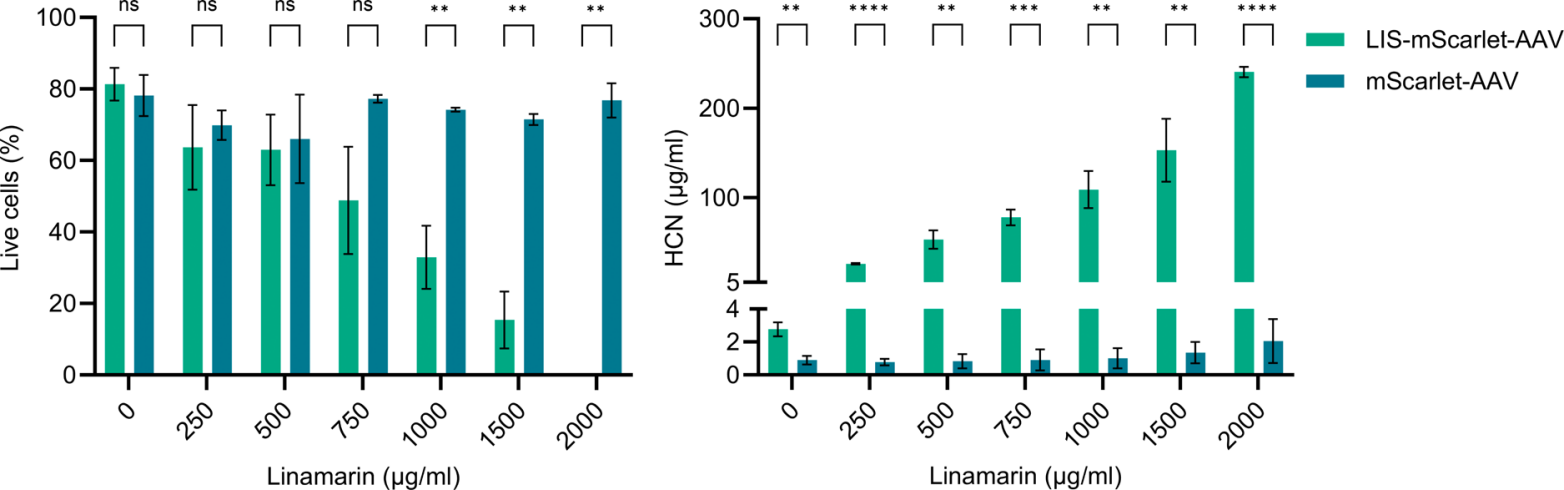
Targeted cancer cell killing using the linamarase suicide gene. Live cell quantification by flow cytometry with Zombie violet staining and HCN production analysis after delivering the linamarase suicide gene and incubation of cells with different concentrations of linamarin. SpyT453-AAVs (2.4 × 10^8^ vg/mL, MOI: 5.9 × 10^3^) carrying either linamarase-mScarlet fusion or mScarlet gene alone (control) were preincubated with 10 nM SpyC-DARPin_EGFR_ and used to transduce A-431 cells. Statistical significance is indicated as follows: ns (*p* > 0.05), **­(*p* ≤ 0.01), ***­(*p* ≤ 0.001), ****­(*p* ≤ 0.0001). Bars represent means ± SD of *n* = 3 biological replicates.

## Discussion

Recombinant adeno-associated viral vectors have emerged as the vector of choice for *in vivo* gene delivery, but their widespread adoption faces key challenges. These include limited tissue specificity, manufacturing challenges, and the presence of neutralizing antibodies in patients.^63^ Numerous studies have proposed innovative solutions to address these hurdles, with modular targeting platforms showing promise for improved specificity and simplified production for discovery and preclinical studies.

In this study, we developed a modular AAV-based retargeting platform, for the first time leveraging the SpyTag/SpyCatcher technology via genetic integration for the rapid and flexible postassembly functionalization of AAV capsids with targeting ligands. By inserting SpyTags into defined positions on the AAV2 capsid, we created a universal vector scaffold that can be redirected to diverse targets by coupling them to SpyCatcher-fusion ligands. We demonstrated this modularity by equipping the vector with different SpyCatcher-DARPin fusion proteins specific for EGFR, EpCAM, and HER2/ErbB2, thereby achieving efficient and specific transduction of corresponding cancer cell lines. Additionally, we validated the platform’s therapeutic functionality by delivering a suicide gene, which induced selective cell death upon prodrug activation.

Our approach builds on earlier strategies for modular AAV targeting, including the split-intein-based approach,^21^ where the surface of fully assembled AAV capsids carrying a C-intein at the N-terminus of VP2 were equipped with N-intein-fused single-chain variable fragments (scFvs) or DARPins and the click-chemistry-based approach for SpyTag-display on AAV.^29^ The introduction of unnatural amino acids into AAV capsids and subsequent functionalization with ligands via click-chemistry was shown to allow the alteration of vector tropism with site-specific single-residue targeting, high versatility, and a high coupling efficiency with predictable stoichiometry.^29,64–66^ In contrast, our system relies solely on genetic incorporation of the SpyTag into the capsid, providing a simpler and more direct route to postassembly vector modification. Our system offers a “plug and play” modular characteristic without the need for complex chemical reagents and easy scalability. We identified capsid positions 453 and 587 as suitable for SpyTag insertion and showed that incorporation into all three capsid proteins led to more efficient surface display than selective VP2-only modification, likely due to better incorporation ratios and capsid assembly dynamics.

Although Western blot analysis suggested that only a subset of capsid proteins displayed accessible SpyTags in the native conformation, even limited functionalization was sufficient to achieve high transduction efficiencies. This suggests that only a few accessible ligands may be needed for effective receptor-mediated entry. Functionalization could potentially be increased by the introduction of linker sequences. Flexible linkers consisting mainly of glycine and serine residues are frequently used to maximize flexibility, surface exposure, and independent folding of fusion proteins and could increase conformational freedom in SpyTag exposure and accessibility. More rigid structures such as helical or proline rich linkers could increase spatial separation without interfering with folding.^67^ Additionally, modifying flanking residues to enhance externalization, as suggested in previous studies^68^ could increase functionalization. Simultaneously, this low functionalization enables the application of the system with a minimum of nonhuman-derived protein adapters, thereby lowering the risk of immunogenic reaction. Furthermore, the use of minimally modified rAAV2 capsids ensures compatibility with standard production workflows, while the SpyTag/SpyCatcher system’s robustness across physiological buffers simplifies application in biologically relevant settings. Compared to intein-based or chemically modified systems, our approach avoids the need for reducing agents or nonphysiological conditions and expands the range of compatible ligands, including disulfide-rich or structurally complex proteins.

The successful coupling of a newly engineered SpyCatcher variant (SpyCatcher003Δ) further demonstrates the flexibility of the system and points to opportunities for optimizing binding kinetics and efficiency through next-generation SpyTag/SpyCatcher pairs. The modular postassembly nature of this platform also enables future exploration of complex ligands such as scFvs, nanobodies, Fab’ fragments, or native ligands as established in the field^21,27,29^ or multispecific targeting strategies, for example, by coupling bispecific DARPins^69^ or ligand mixtures to enhance cell-type specificity and therapeutic precision. For therapeutic applications, the SpyT587-AAV variant generated in this study holds special potential due to its lower off-target background transduction.

Beyond its therapeutic potential, this platform offers a valuable tool for the rapid screening and preclinical evaluation of targeted AAVs. The ability to use a single capsid scaffold and test multiple targeting ligands in parallel accelerates the discovery pipeline and reduces the need for repeated vector engineering and validation. Furthermore, the use of a single capsid for various targets could justify the generation of a producer cell line, thereby saving costs and facilitating the production process. While the bacterial origin of SpyCatcher may raise legitimate concerns regarding potential immunogenicity, available data from *in vitro* and *in vivo* studies to date indicate that SpyCatcher itself is only weakly immunogenic when administered as a soluble protein.^27,70–72^ Janitzek *et al.* reported little to no detectable humoral or cellular *anti*-SpyCatcher response in mice. In contrast, a specific *anti*-SpyCatcher antibody response has been observed when SpyCatcher was presented in a highly multivalent format on virus-like particles or carrier proteins in conjunction with adjuvants.^72^ While the degree of surface exposure of SpyTag on AAV in our system is limited, this is an important parameter for future translational work and necessitates a more comprehensive assessment of immunogenicity, including *anti*-SpyCatcher responses, and systematic delivery of functionalized SpyTag-AAV, will be essential in subsequent *in vivo* studies.

In conclusion, we present a genetically encoded, modular AAV platform that enables postassembly functionalization via the SpyTag/SpyCatcher system. This strategy allows for flexible, efficient redirection of AAVs to various cellular targets using interchangeable ligands, supporting both therapeutic applications and preclinical vector discovery with a minimal need for capsid redesign.

## Materials and Methods

### Cloning of Plasmids

Plasmids used and generated in this study are listed in Table S8 and their amino acid sequences are depicted in Table S9. Gene sequences were amplified using polymerase chain reaction (PCR) and assembled by Gibson cloning^73^ or restriction enzyme cloning. Mutations and SpyTag sequences were introduced using oligonucleotides and polymerase chain reaction (PCR).

### Protein Production and Purification

For the production of all SpyCatcher-DARPin proteins, the respective plasmids were transformed into *E. coli* BL21 Star (DE3) (Thermo Fisher Scientific, Cat. No. C601003). Transformed bacteria were selected in LB medium supplemented with ampicillin (100 μg/mL) and grown at 37 °C and 150 rpm to an OD_600_ of 0.7, at which expression was induced with 1 mM isopropyl-β-d-thiogalactopyranoside (IPTG). After incubation at 30 °C and 150 rpm for 4 h, bacteria were harvested by centrifugation at 6000*g* for 10 min. The cell pellet was resuspended in lysis buffer [50 mM NaH_2_PO_4_, 300 mM NaCl, 10 mM imidazole, pH 8.0], shock-frozen in liquid nitrogen, and stored at −80 °C. For purification, frozen pellets were thawed at 37 °C in a water bath and lysed by sonication (Bandelin Sonopuls HD3100, 10 min with 60% amplitude, 0.5 s pulse, and 1 s pause intervals). Following clarification of the lysate by centrifugation at 30,000*g* for 30 min at 4 °C, the supernatant was loaded onto a gravity flow column with Ni-NTA Superflow agarose (Qiagen, Cat. No. 30430). The column was washed with 20 column volumes (CV) of wash buffer [50 mM NaH_2_PO_4_, 300 mM NaCl, 20 mM imidazole, pH 8.0], and the purified protein was eluted in 6 CV elution buffer [50 mM NaH_2_PO_4_, 300 mM NaCl, 250 mM imidazole, pH 8.0]. Afterward, the purified protein was dialyzed against PBS [2.7 mM KCl, 1.5 mM KH_2_PO_4_, 8.1 mM Na_2_HPO_4_, and 137 mM NaCl, pH 7.4] using 10 kDa MWCO dialysis tubing (Thermo Fisher Scientific, Cat. No. 88243). Protein aliquots were shock-frozen in liquid nitrogen and stored at −80 °C.

### Protein Characterization

Protein identity and purity was analyzed by sodium dodecyl sulfate-polyacrylamide gel electrophoresis (SDS-PAGE) using 10% gels followed by Coomassie staining. As a protein size marker, a PageRuler prestained protein ladder (Thermo Fisher Scientific, Cat. No. 26616) was used. Protein concentrations were determined by Bradford assay (Bio-Rad, Cat. No. 500-0006) using bovine serum albumin (Sigma-Aldrich, Cat. No. 05479) as the standard.

### Cell Culture

HEK-293T (German Collection of Microorganisms and Cell Cultures (DSMZ), Cat. No. ACC 635) and A-431 (DSMZ, Cat. No. ACC 91) cells were cultivated in Dulbecco’s modified Eagle’s medium (DMEM) (PAN Biotech, Cat. No. P04-03550), supplemented with 10% (v/v) fetal bovine serum (FBS) (PAN Biotech, Cat. No. P30-3602, Lot. No. P150702), 100 U/mL of penicillin, and 100 μg/mL of streptomycin. SK-OV-3 cells (ATCC, Cat. No. HTB-77) were maintained in McCoy’s 5A medium (Sigma-Aldrich, Cat. No. M8403) supplemented with 10% (v/v) FBS, 2 mM l-glutamine (Thermo Fisher Scientific, Cat. No. 25030-024), 100 U/mL penicillin, and 100 μg/mL streptomycin. MDA-MB-453 cells (ATCC, Cat. No. HTB-131) were cultivated in RPMI 1640 medium (Thermo Fisher Scientific, Cat. No. 61870-010) supplemented with 10% (v/v) FBS, 100 U/mL penicillin, and 100 μg/mL streptomycin. CHO-K1 cells were maintained in Ham’s F-12 medium (Thermo Fisher Scientific, Cat. No. 15172529) supplemented with 10% (v/v) FBS, 2 mM l-glutamine, 100 U/mL penicillin, and 100 μg/mL streptomycin. All cells were cultivated at 37 °C in a humidified atmosphere containing 5% CO_2_ and passaged upon reaching approximately 80% confluence.

### AAV Production and Purification

AAV vectors were produced using the adenovirus helper-free packaging system.^74^ For the production of SpyTag-AAVs, HEK-293T cells were transfected with an AAV2 rep–cap plasmid (Cell Biolabs, Cat. No. VPK-402), an adenovirus helper plasmid (Cell Biolabs, Cat. No. VPK-402), and a self-complementary vector plasmid pCMV-mScarlet (or derivatives of it) (a gift from Dirk Grimm) in an equimolar ratio. For AAV production, 8 × 10^6^ HEK-293T cells were seeded per 15 cm cell culture dish and 24 h later, transfected with 60 μg of total plasmid DNA combined with 200 μg of polyethylenimine (PEI, MW 25,000, Polysciences, Cat. No. 23966) in 3 mL OptiMEM (Thermo Fisher Scientific, Cat. No. 22600-134). After 72 h, AAVs were precipitated from the supernatant using polyethyleneglycol (PEG). To this end, the supernatant was collected and combined with a 40% PEG 8000 solution [40% (w/v) PEG 8000, 0.41 M NaCl, pH 7.4] to a final PEG concentration of 8% and incubated at 4 °C with slight agitation overnight. AAVs were then harvested by centrifugation at 4 °C and 2828*g* for 15 min. The pellet was resuspended in an appropriate amount of sterile PBS (Thermo Fisher Scientific, Cat. No. 14190094) and filtered through a 0.45 μm polyvinylidene difluoride (PVDF) syringe filter. Aliquots were shock-frozen in liquid nitrogen and stored at −80 °C.

### AAV Characterization by Western Blot

AAV preparations were analyzed for the presence of capsid proteins and SpyCatcher coupling by Western blotting against the viral capsid proteins. Depending on the condition, AAVs were either combined with SpyCatcher-DARPin protein, denatured at 98 °C for 10 min, and then combined with SpyCatcher-DARPin protein, or combined with PBS and incubated at room temperature for 1 h. Samples were then mixed with 5x SDS loading buffer [50% (v/v) glycerol, 0.3125 M Tris–HCL pH 6.8, 0.05% (w/v) bromophenol blue, 10% (w/v) SDS, 12.5% (v/v) 2-mercaptoethanol] and incubated at 70 °C for 10 min. AAV and protein concentrations are listed in Tables S1 and S2. After the samples were separated by SDS-PAGE, the proteins were blotted onto a methanol-activated PVDF membrane. The membrane was then blocked in blocking buffer [PBS containing 5% (w/v) low fat milk powder (Carl Roth, blotting grade, Cat. No. T145.3)] for 1 h at room temperature before incubation with *anti*-AAV VP1/VP2/VP3 B1 antibody (Progen, Cat. No. 65158) diluted 1:100 in binding buffer [PBS containing 2.5% (w/v) milk powder) at 4 °C overnight. SpyCatcher protein was detected using *anti*-His antibody (Merck, Cat. No. 70796-3) diluted to 0.1 μg/mL in binding buffer. Subsequently, the membrane was washed with PBS-T [PBS supplemented with 0.05% (v/v) Tween-20] and incubated with a secondary *anti*-mouse horseradish peroxidase (HRP)-conjugated antibody (Amersham Cytiva, Cat. No. NA931) diluted 1:5000 in binding buffer. After washing with PBS-T, either the Pierce ECL Western Blotting Substrate (Thermo Fisher Scientific, Cat. No. 32109) (Figure 2, upper panel and Figure S3B) or SuperSignal West Femto Maximum Sensitivity Substrate (Thermo Fisher Scientific, Cat. No. 34094) (Figure 2, lower panel, Figures S3A and S5) was added and chemiluminescence was imaged using the ImageQuant LAS 4000 mini System (GE Healthcare).

Western blot bands were quantified in Fiji.^75^ Images were converted to an 8 bit grayscale. Individual rectangular ROIs were applied to all lanes; lane profiles were generated using the Gel Analyzer and peak areas were integrated after baseline subtraction. All quantification was performed on the original, unadjusted images.

### AAV Quantification by qPCR

The genomic titer of AAV vectors was determined by quantitative real-time PCR (qPCR). AAV samples were DNase I (New England Biolabs, Cat. No. M0303S) digested at 37 °C for 30 min. Then, serial dilutions of AAVs and of the vector plasmid CMV-mScarlet as standard were prepared, and oligonucleotides 5′-TGCCCAGTACATGACCTTATGG-3′ and 5′-GAAATCCCCGTGAGTCAAACC-3′ were used to amplify a 134 bp fragment from the CMV promoter using the PowerTrack SYBR Green Mastermix (Thermo Fisher Scientific, Cat. No. A46109). The qPCR was performed on a CFX384 thermocycler (Bio-Rad) with the following temperature protocol: 3 min at 95 °C followed by 40 cycles of 15 s at 95 °C, 30 s at 60 °C, read plate, and finally followed by a melting curve.

### AAV Transduction Experiments

For transduction experiments, 4000 cells were seeded per well of a 96-well plate. After 24 h, SpyTag-AAVs were mixed with SpyCatcher-DARPin protein in transduction medium (DMEM, supplemented with 10% (v/v) FBS, 100 U/mL penicillin, 100 μg/mL streptomycin, and 10 mM HEPES (Thermo Fisher Scientific, Cat. No. 15630056)) and incubated at 37 °C for 1 h or as indicated. The final transduction mix consisted of 10 μL of appropriately diluted AAV and 10 μL of appropriately diluted SpyCatcher-DARPin protein, filled up to a final volume of 100 μL with transduction medium. Following complete removal of medium from the cells, 100 μL of transduction mix was added per well and cells were incubated at 37 °C in a humidified atmosphere containing 5% CO_2_ for 48–72 h.

For linamarin experiments, serial dilutions of linamarin (α-hydroxyisobutyronitrile β-d-glucopyranoside, Merck, Cat. No. 68264) in ddH_2_O were prepared. 20 μL of linamarin dilutions was added to cells 24 h after transduction (final volume: 120 μL per well). The 96-well plate was then sealed with qPCR covering film (Greiner, Cat. No. 676040) in order to prevent gaseous HCN from evaporating or spreading to other wells and incubated at 37 °C in a humidified atmosphere containing 5% CO_2_ for 48 h.

### Dialysis of SpyC-DARPin_EGFR_-Coupled SpyT453-AAV

SpyT453-AAV was incubated with 5 nM of SpyC-DARPin_EGFR_ in a total volume of 70 μL for 2 h at RT. The coupling mixture was applied to a 1000 kDa MWCO dialysis membrane (Biotech CE Dialysis Tubing 1000KD, Replingen, Cat. No. 131486) and dialyzed against 1 L of 1x PBS for 4 days at 4 °C (PBS was exchanged on day 3). For Western blot analysis, 5 μL of predialysis input mixture and 5 μL of the dialyzed sample were each diluted in 15 μL 1x PBS, mixed with 5x SDS loading buffer, and heated at 95 °C for 5 min. 10 μL was loaded per lane on 15% acrylamide gels. SDS-PAGE and Western blotting were performed as described above. SpyT-AAV and SpyC-DARPin_EGFR_ concentrations are listed in Table S3.

For transduction analysis, the dialyzed sample was diluted 1:800 in transduction medium, and 100 μL of the diluted sample was added to 4000 A-431 cells per well. Downstream processing was performed as described in other transduction experiments.

### Flow Cytometry

For analysis by flow cytometry, cells were washed with PBS and detached by the addition of 50 μL of trypsin/EDTA solution (PAN Biotech, Cat. No. P10-023100) per well. Then, 200 μL of FACS buffer (PBS supplemented with 2% (v/v) FBS) was added per well, cells were resuspended and analyzed by flow cytometry. For linamarin experiments with live/dead analysis, cells were stained using the Zombie Violet Fixable Viability Kit (Biolegend, Cat. No. 423113) according to the standard cell staining protocol. In brief, following resuspension in FACS buffer, cells were washed with PBS and resuspended in 100 μL of staining solution containing 1:500 Zombie Violet dye in PBS. After incubation for 30 min at RT in the dark, cells were washed with FACS buffer and finally resuspended in 220 μL of FACS buffer. Cells were analyzed for mScarlet transgene expression and Zombie Violet staining using an Attune NxT flow cytometer (Thermo Fisher Scientific). BFP and Zombie Violet were excited with a 405 nm laser and detected using a 440/50 nm filter, while mScarlet was excited using a 561 nm laser and detected using a 620/15 nm filter. Cellular autofluorescence was measured in the unused BFP channel for experiments without Zombie Violet or using a 637 nm laser and 670/14 nm emission filter for experiments with Zombie Violet staining. Flow cytometry data were analyzed using FlowJo (v10.9.0, Becton Dickinson) with the gating strategy depicted in Figures S8 and S9.

### Head Space-Solid Phase Microextraction Gas Chromatography Mass Spectrometry (HS-SPME GCMS)

Samples were prepared for GCMS analysis by diluting 90 μL of sample cell culture supernatant in 810 μL of transduction medium. The quantification of HCN in samples was performed with a single quadrupole GCMS system QP2010 SE (Shimadzu, Japan) equipped with a PAL autosampler AOC 5000 (CTC Analytics, Zwingen, Switzerland). The HS-SPME technique was used for sample preparation. For this purpose, 200 ± 1 mg of Na_2_SO_4_, 400 μL of the sample or standard solution, 20 μL of a 3 μg/mL CD_3_CN solution as an internal standard, and 75 μL of 85% phosphoric acid were added to each HS vial and immediately sealed with PTFE/silicone septa. HS-SPME was achieved on a CBX/PDMS fiber (75 μm thickness) with 10 min extraction time at 35 °C with agitation followed by thermal desorption directly into the splitless injector at 250 °C. After each run, the fiber was conditioned at 250 °C for 30 min. A He column flow of 1 mL/min was realized, which corresponds to a linear velocity of 36 cm/s. The following temperature program was carried out on a DB-FFAP column (30 m × 0.25 mm, thickness 0.25 μm): start temperature 40 °C for 8 min, heating with 40 K/min to 240 °C, and 5 min hold time at a final temperature of 240 °C. The mass spectra were recorded in SIM mode at *m*/*z* of 27 and 44. The MS interface was set to 250 °C and the ion source temperature to 200 °C. Internal standard calibration was done between 0 and 25 μg/mL HCN, achieving correlation coefficients of 99.95%.

### Statistical Analysis

For the analysis of differences between linamarase-mScarlet-carrying AAV and mScarlet control AAV, unpaired two-sided *t* tests under no assumption of consistent standard deviations with correction for multiple comparisons (Holm–Sidak method) were performed using GraphPad Prism.

### Software

Flow cytometry data were analyzed with FlowJo (v10.9.0, Becton, Dickinson and Company). *In silico* cloning and sequence alignments were performed using SnapGene (v8.0.3, SnapGene Software). Data were analyzed and plotted using GraphPad Prism (v10.2.0, GraphPad Software). Western blots were quantified using Fiji (ImageJ).^75^ Figure 1 was created with BioRender.

## Supplementary Material



## Notes

Coomassie staining of SpyCatcher001Δ-DARPin_EGFR_ purification; transduction tests of wtAAV2 on A-431 cells and SpyT453-AAV on CHO–K1 cells with and without SpyCatcher-DARPin_EGFR_; dialysis of Spy- Catcher-AAV complexes; alignment of SpyCatcher variants; Coomassie staining of SpyCatcher003-DARPin_EGFR_, SpyCatcher001-DARPin_EGFR_, and SpyCatcher003Δ-DARPin_EGFR_ purification; Coomassie staining of SpyCatcher-DARPin_EpCAM_, SpyCatcher-DARPin_HER2_, and SpyCatcher-DARPin_CD4_ purification; transduction tests in A-431 cells and Western Blot of SpyT453-AAV carrying linamarase-mScarlet; flow cytometry gating strategy of SpyT-AAV transduced samples; flow cytometry gating strategy of SpyT-AAV transduced and live/dead stained samples; AAV and protein concentrations corresponding to Figure 2; relative capsid protein ratios corresponding to Figure 2; AAV and protein concentrations corresponding to Figure S3; AAV and protein concentrations corresponding to Figure S6; linamarin conversion efficiency; AAV production titers; overview of AAVs; plasmids used in this study; and nucleic acid sequences of plasmids (PDF)

## ∇

Prolific Machines, Emeryville, CA 94608, United States
