# Supplementary material for: Genetically Encoded SpyTag Enables Modular AAV Retargeting via SpyCatcher-Fused Ligands for Targeted Gene Delivery
Source: ACS Synth Biol. 2025 Dec 22;15(1):149–60. doi: 10.1021/acssynbio.5c00565 (PMC12814547; doi:10.1021/acssynbio.5c00565)
Supplement: Supplementary file 1 [file sb5c00565_si_001.pdf]

Supporting Information

Genetically encoded SpyTag enables modular AAV retargeting via SpyCatcher-fused ligands for targeted gene delivery

Authors: Anja Armbruster[1,2,3], Maximilian Hörner[2,3,4], Hanna J Wagner[2,3], Claudia Fink-Straube[1], Wilfried Weber[1,5,*]

[1] INM – Leibniz Institute for New Materials, 66123 Saarbrücken, Germany

[2] Signalling Research Centres BIOSS and CIBSS, University of Freiburg, 79104 Freiburg i. Br., Germany

[3] Faculty of Biology, University of Freiburg, 79104 Freiburg i. Br., Germany

[4] Current address: Prolific Machines, Emeryville, CA 94608, United States

[5] Saarland University, Department of Materials Science and Engineering, Saarbrücken 66123, Germany

[*] Email: Wilfried.Weber@leibniz-inm.de

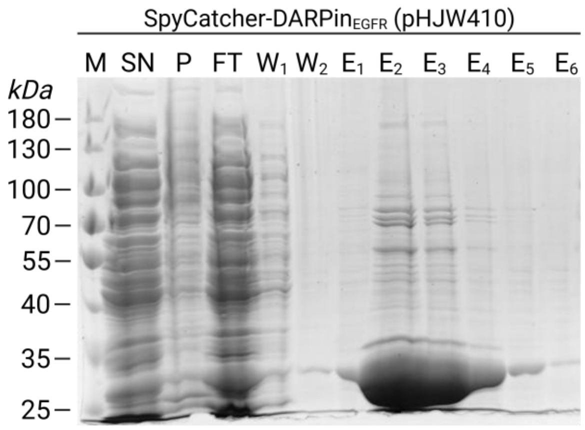

Figure S1: Analysis of SpyC-DARPin$_{EGFR}$ purification by SDS-PAGE and Coomassie staining.

SpyCatcher-DARPin$_{EGFR}$ was produced in *E. coli* from plasmid pHJW410 and purified by IMAC. Samples from different purification steps were subjected to analysis by SDS-PAGE followed by Coomassie staining. M, protein size marker; SN, soluble fraction of lysate; P, insoluble fraction of lysate; FT, flow through; W, wash; E, elution after purification. Calculated SpyCatcher-DARPin$_{EGFR}$ mass: 27.0 kDa.

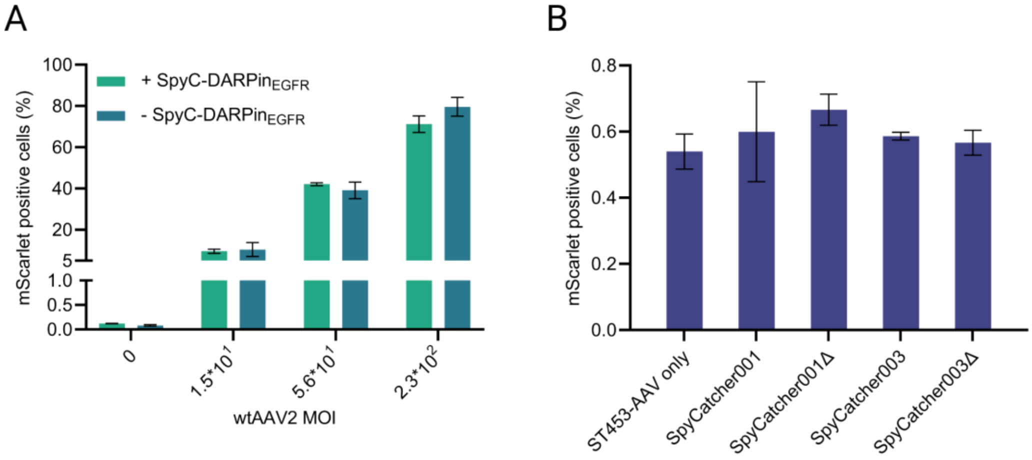

Figure S2: Testing SpyC-DARPin-directed transduction for specificity.

A: wtAAV2 carrying mScarlet was serially diluted (starting form $9.3*10^6$ vg/ml, then diluted serially 1:4) and incubated with 10 nM of SpyC-DARPin$_{EGFR}$ for 1 h at 37°C prior to transduction of A-431 cells. **B:** SpyT453-AAV ($4.0*10^8$ vg/ml, MOI: $1.0*10^3$) was incubated with SpyCatcher-DARPinEGFR variants for 1 h at 37 °C prior to transduction of CHO-K1 cells. For both experiments, transduction efficiency was analyzed after 48 h by quantifying mScarlet-positive cells by flow cytometry. Bars represent means ± SD of n=3 biological replicates.

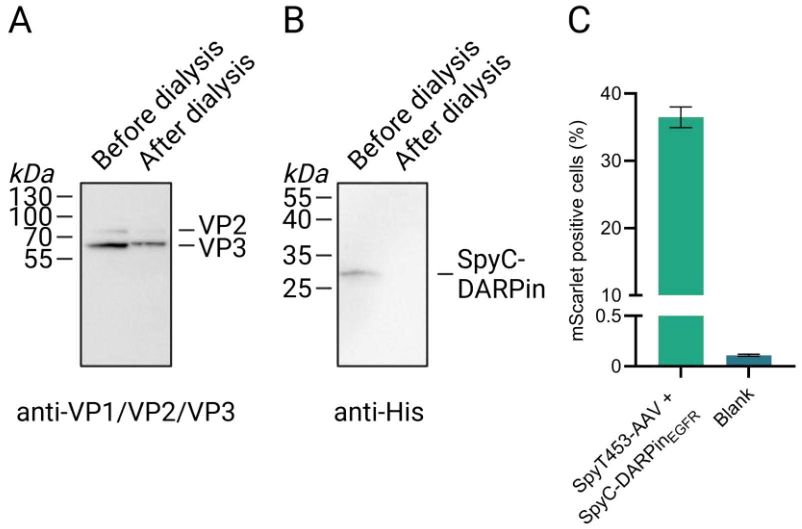

Figure S3: Removal of excess unbound SpyC-DARPin from SpyT-AAV by dialysis.

A and B: Western blot analysis of SpyC-DARPin$_{EGFR}$-coupled SpyT453-AAV before and after dialysis. SpyT453-AAV was coupled with 5 nM of SpyC-DARPin$_{EGFR}$ and subsequently dialyzed against PBS for 4 days. At this loading, only VP3 is visible. **A** depicts anti VP1/VP2/VP3 blot (SpyT453-AAV), **B** depicts anti-His blot (SpyC-DARPin$_{EGFR}$). SpyT-AAV and SpyC-DARPin$_{EGFR}$ concentrations are listed in Table S3. **C:** Transduction of A-431 with dialyzed SpyT453-AAV-SpyC-DARPin$_{EGFR}$ complexes (5 µl of 1:40 diluted mix per well). Transduction efficiency was analyzed after 48h by quantifying mScarlet positive cells by flow cytometry. Bars represent means ± SD of n=3 biological replicates. Untransduced cells served as blank control.

```
SpyCatcher001    1    MAGVDTLSGLSSEQGQSGDMTIEEDSATHIKFSKRDEDGKELAGATMELRDSSGKTISTWISDGQVKDFY
SpyCatcher001Δ   1    ---------------------MSGDSATHIKFSKRDEDGKELAGATMELRDSSGKTISTWISDGQVKDFY
SpyCatcher003    1    --MVTTLSGLSGEQGPSGDMTTEEDSATHIKFSKRDEDGRELAGATMELRDSSGKTISTWISDGHVKDFY
SpyCatcher003Δ   1    ---------------------MSGDSATHIKFSKRDEDGRELAGATMELRDSSGKTISTWISDGHVKDFY

SpyCatcher001    71   LYPGKYTFVETAAPDGYEVATAITFTVNEQGQVTVNGKATKGDAHI
SpyCatcher001Δ   50   LYPGKYTFVETAAPDGYEVATAITFTVNEQGQVTVNG---------
SpyCatcher003    69   LYPGKYTFVETAAPDGYEVATPIEFTVNEDGQVTVDGEATEGDAHT
SpyCatcher003Δ   50   LYPGKYTFVETAAPDGYEVATPIEFTVNEDGQVTVDG---------
```

Figure S4: Generation of SpyCatcher003Δ by sequence comparison with SpyCatcher001Δ.

SpyCatcher003Δ was generated after comparing the protein sequences of SpyCatcher001 and SpyCatcher001Δ, identifying the truncated regions in SpyCatcher001Δ and applying the same truncations to the parental SpyCatcher003 sequence to finally yield SpyCatcher003Δ. Consensus sequence is marked blue. Alignment was generated using MUSCLE in SnapGene.

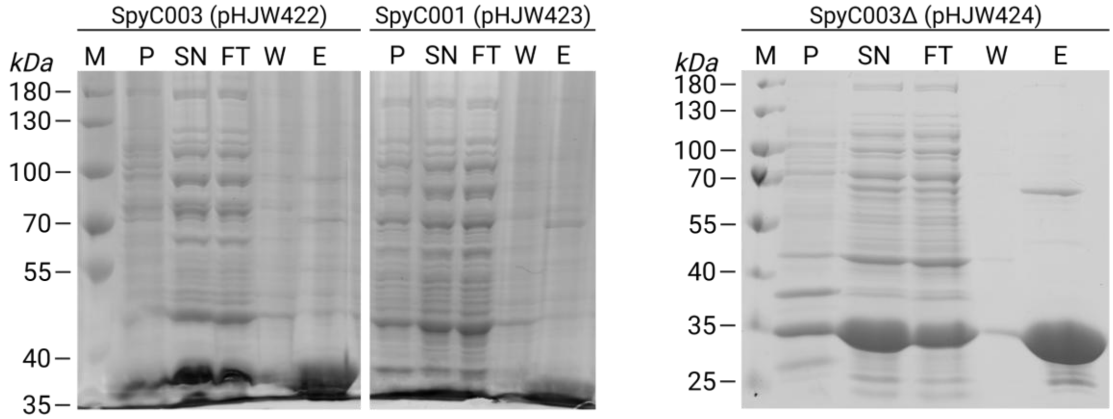

Figure S5: Analysis of SpyCatcher variants purification by SDS-PAGE and Coomassie staining.

SpyCatcher003-DARPin$_{EGFR}$, SpyCatcher001-DARPin$_{EGFR}$, SpyCatcher003Δ-DARPin$_{EGFR}$ were produced in *E. coli* from plasmids pHJW422, pHJW423 and pHJW424, respectively and purified by IMAC. Samples from different purification steps were subjected to analysis by SDS-PAGE followed by Coomassie staining. M, protein size marker; SN, soluble fraction of lysate; P, insoluble fraction of lysate; FT, flow through; W, wash; E, elution after purification. Calculated masses: SpyCatcher003-DARPin$_{EGFR}$, 30.2 kDa; SpyCatcher001-DARPin$_{EGFR}$, 30.5 kDa; SpyCatcher001-DARPin$_{EGFR}$, 27.3 kDa.

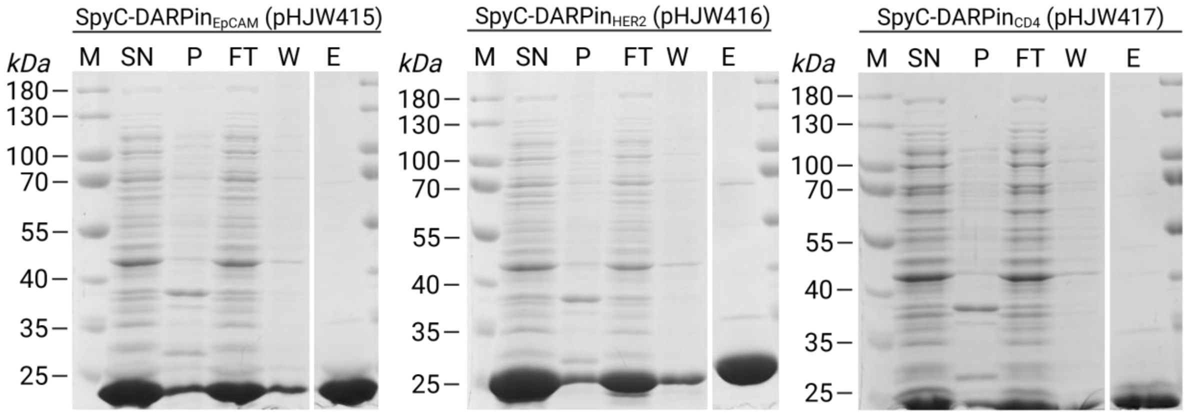

Figure S6: Analysis of SpyCatcher-DARPin$_{EpCAM}$, SpyCatcher-DARPin$_{HER2}$ and SpyCatcher-DARPin$_{CD4}$ purification by SDS-PAGE and Coomassie staining.

SpyCatcher-DARPin$_{EpCAM}$, SpyCatcher-DARPin$_{HER2}$ and SpyCatcher-DARPin$_{CD4}$ were produced in *E. coli* from plasmids pHJW415, pHJW416 and pHJW417, respectively and purified by IMAC. Samples from different purification steps were subjected to analysis by SDS-PAGE followed by Coomassie staining. M, protein size marker; SN, soluble fraction of lysate; P, insoluble fraction of lysate; FT, flow through; W, wash; E, elution after purification. Calculated masses: SpyCatcher-DARPin$_{EpCAM}$, 27.5 kDa; SpyCatcher-DARPin$_{HER2}$, 27.4 kDa, SpyCatcher-DARPin$_{CD4}$, 23.7 kDa.

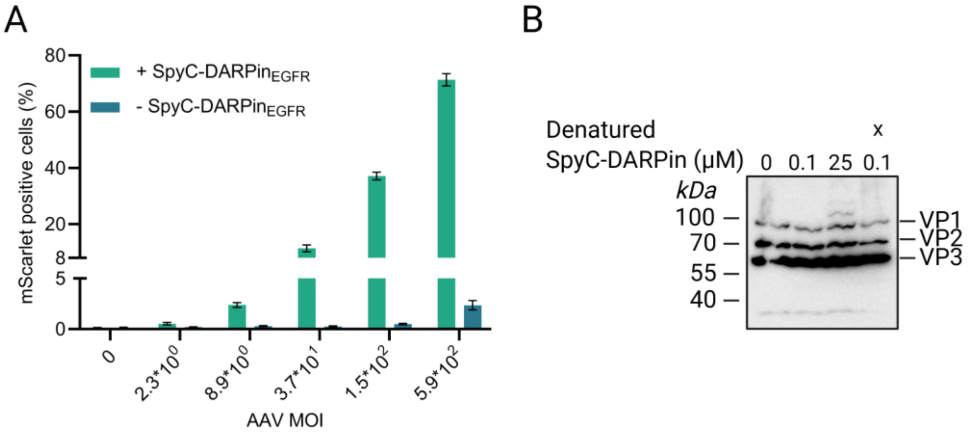

Figure S7: Characterization of LIN-mScarlet-AAV

A: SpyT453-AAV (starting from $2.3*10^7$ vg/ml, then diluted serially 1:4) was incubated with or without 100 nM of SpyCatcher-DARPin$_{EGFR}$ for 1 h at 37 °C prior to transduction of A-431 cells Transduction efficiency was analyzed after 48 h by quantifying mScarlet-positive cells by flow cytometry. Bars represent means ± SD of n=3 biological replicates. **B:** Western blot analysis of PEG-precipitated viral capsid proteins VP1, VP2 and VP3 of SpyT453-AAV carrying linamarase-mScarlet from cell culture supernatant. Numbers indicate the concentration of SpyCatcher-DARPin$_{EGFR}$ protein during incubation. "x" denotes AAV denaturation by boiling at 98 °C for 10 min before SC-DARPin coupling. SpyT-AAV and SpyC-DARPin$_{EGFR}$ concentrations are listed in Table S3.

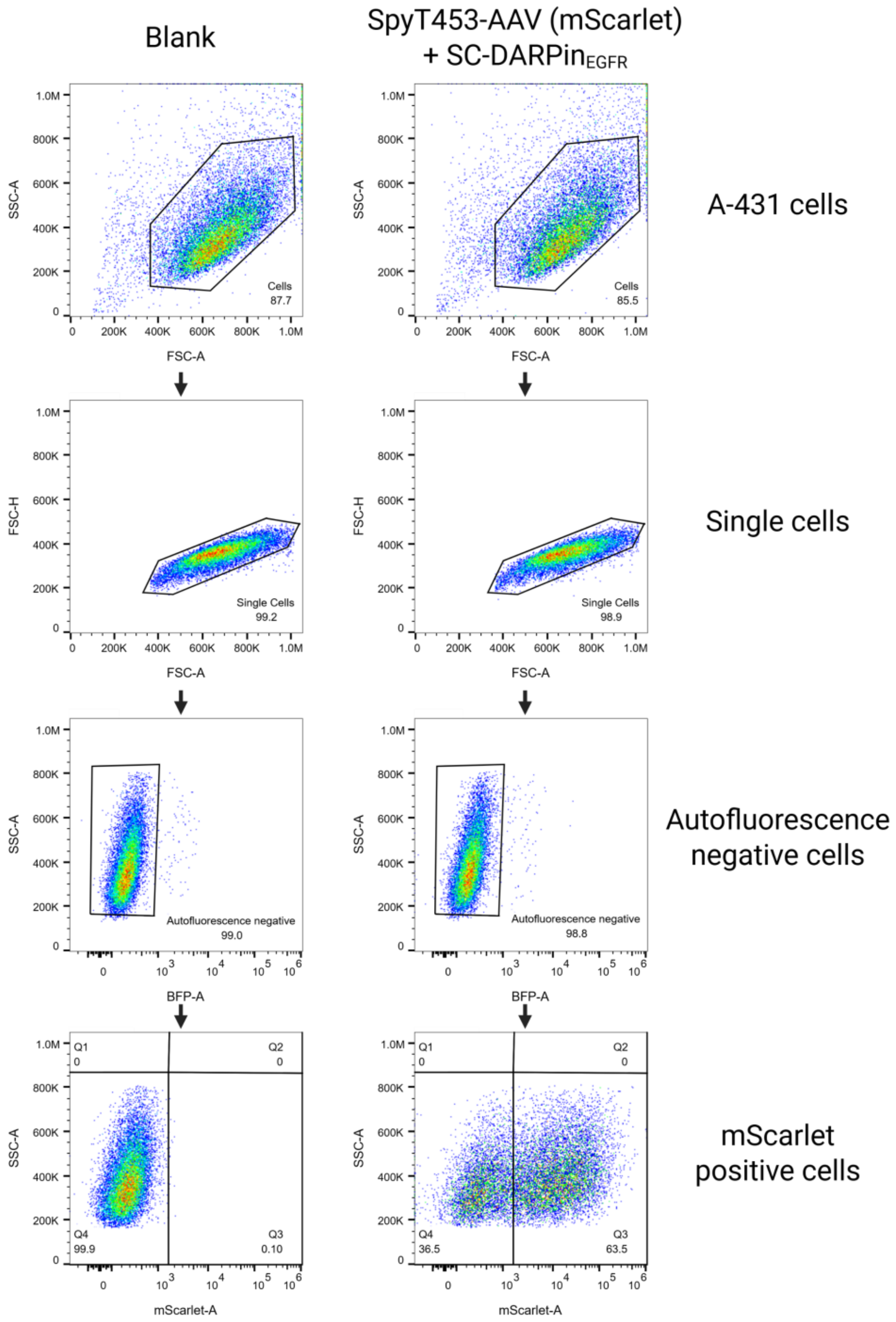

Figure S8: Gating strategy of AAV transduction experiments analyzed by flow cytometry.

The gating strategy of representative samples from experiments performed with SpyTag-AAVs on various cancer cell lines (depicted here: A-431 cells) is shown. First, cells were gated based on FSC-A and SSC-A signal, then doublets were excluded based on FSC-H and FSC-A signal. Autofluorescent cells were excluded based on BFP-A signal and mScarlet positive cells were gated based on untransduced cells (Blank). Data corresponds to Fig. 3 in the main text.

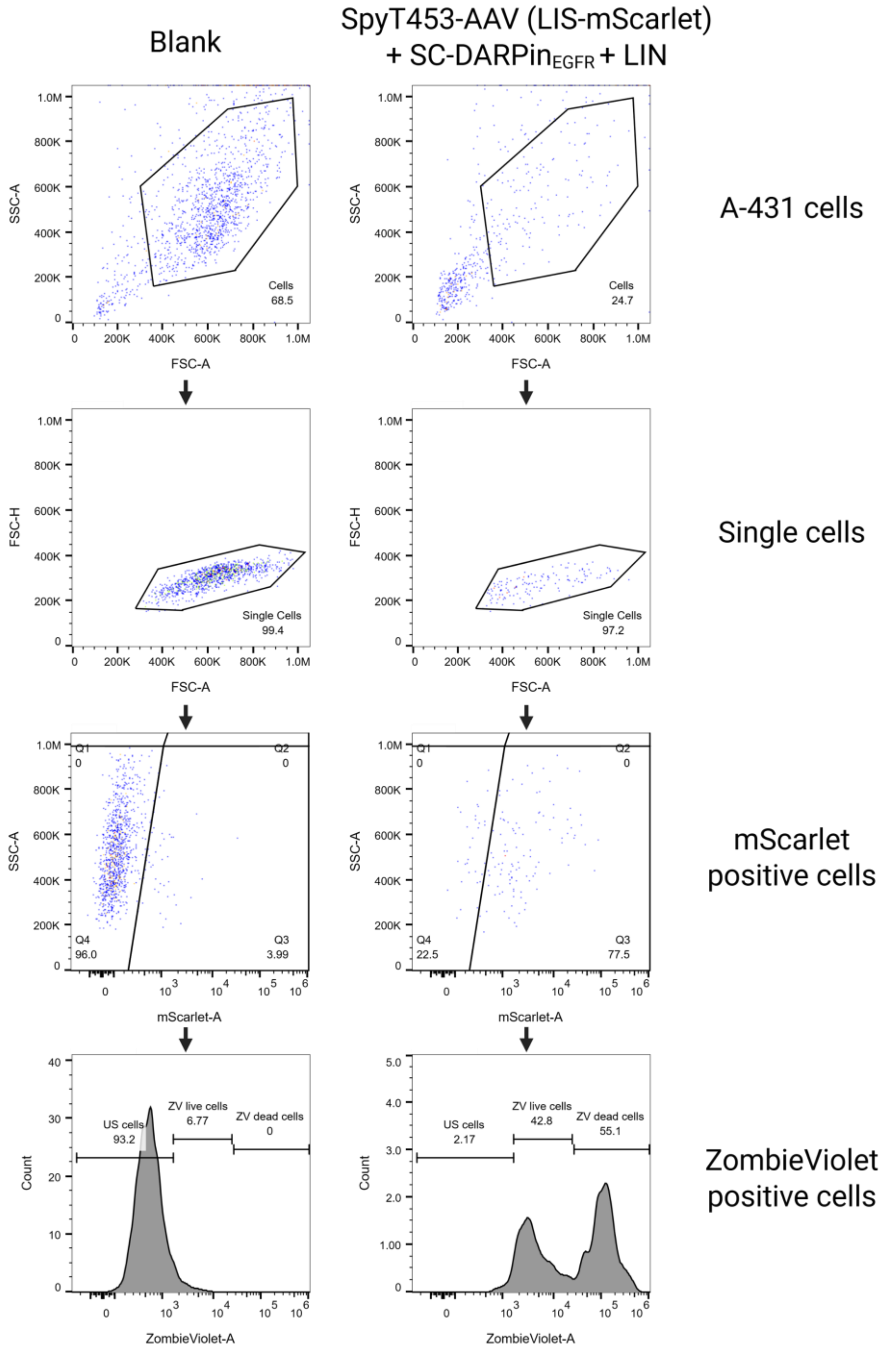

Figure S9: Gating strategy of AAV transduction experiments with live/dead staining analyzed by flow cytometry.

The gating strategy of representative samples from experiments performed with SpyTag-AAVs and live/dead staining of A-431 cells is shown. Cells were gated based on FSC-A and SSC-A signal, before excluding doublets based on FSC-H and FSC-A signal. mScarlet positive cells were gated based on untransduced samples (Blank). Zombie Violet positive cells were gated based on unstained samples (Blank). Data corresponds to Fig. 6 In the main text. Abbreviations: LIN, Linamarin; LIS, Linamarase.

Table S1: SpyT-AAV and SpyC-DARPin$_{EGFR}$ concentrations in the Western blot (Fig. 2).

Concentrations of SpyT-AAV and SpyC-DARPin$_{EGFR}$ during coupling (sample volume: 40 µl) and after loading onto SDS-PAGE (volume: 10 µl). Data corresponds to Fig. 2 in the main text.

	Coupling with SpyC-DARPin$_{EGFR}$		SDS-PAGE and Western blot	
	AAV (vg/ml)	SpyC-DARPin (µM)	AAV (vg)	SpyC-DARPin (nmol)
SpyT587-AAV	$2.5*10^{10}$	42.0	$2.0*10^{8}$	0.336
SpyT453-AAV	$1.1*10^{10}$	42.0	$9.0*10^{7}$	0.336
SpyT587VP2-AAV	$5.3*10^{10}$	42.0	$4.3*10^{8}$	0.336
SpyT453VP2-AAV	$7.3*10^{10}$	42.0	$5.8*10^{8}$	0.336

Table S2: Relative capsid protein ratios of SpyTag-AAVs in the Western blot (Fig. 2)
Quantification of VP1, VP2 and VP3 band intensity by Western blot densitometry for each SpyTag-AAV variant and calculation of relative ratios of VP2 and VP3 to VP1. Data corresponds to Fig. 2 (upper panel) in the main text.

		SpyT587-AAV	SpyT453-AAV	SpyT587VP2-AAV	SpyT453VP2-AAV
Area	VP1	907	2047	5464	598
	VP2	2407	2179	294	0
	VP3	11394	10736	10273	12426
VP ratios	VP1/VP1	1	1	1	1
	VP2/VP1	3	1	0	0
	VP3/VP1	13	5	2	21

Table S3: SpyT-AAV and SpyC-DARPin$_{EGFR}$ concentrations in the Western blot (Fig. S3).
Concentrations of SpyT453-AAV and SpyC-DARPin$_{EGFR}$ during coupling and after loading onto SDS-PAGE (volume: 10 µl). Data corresponds to Fig. S3 in the supplementary information.

	Coupling with SpyC-DARPin$_{EGFR}$		SDS-PAGE and Western blot	
	AAV (vg/ml)	SpyC-DARPin (nM)	AAV (vg)	SpyC-DARPin (pmol)
Lane 1	$1.6*10^{10}$	5.0	$3.2*10^{7}$	0.01005

Table S4: SpyT-AAV and SpyC-DARPin$_{EGFR}$ concentrations in the Western blot.(Fig. S6)

Concentrations of SpyT453-AAV (carrying linamarase-mScarlet) and SpyC-DARPin$_{EGFR}$ during coupling (sample volume: 20 µl) and after loading onto SDS-PAGE (volume: 10 µl). Data corresponds to Figure S6 in the supplementary information.

	Coupling with SpyC-DARPin$_{EGFR}$		SDS-PAGE and Western blot	
	AAV (vg/ml)	SpyC-DARPin (µM)	AAV (vg)	SpyC-DARPin (nmol)
Lane 1	$5.5*10^9$	-	$4.4*10^7$	-
Lane 2	$5.5*10^9$	0.1	$4.4*10^7$	0.000807
Lane 3	$5.5*10^9$	25.2	$4.4*10^7$	0.202
Lane 4	$5.5*10^9$	0.1	$4.4*10^7$	0.000807

Table S5: Conversion efficiency of Linamarin into HCN (Fig. 6).

Conversion efficiency of Linamarin into HCN was calculated from mean quantified HCN in linamarase transduced and linamarin treated samples. Data corresponds to Fig. 6 in the main text.

Linamarin (µg/ml)	Linamarin (µmol/ml)	Mean HCN (µg/ml)	Mean HCN (µmol/ml)	Conversion Efficiency (%)
0	0.00	2.78	0.10	
250	1.01	25.85	0.96	94.56
500	2.02	53.07	1.96	97.08
750	3.03	77.83	2.88	94.91
1000	4.04	108.95	4.03	99.65
1500	6.07	153.02	5.66	93.31
2000	8.09	240.58	8.90	110.02
			Mean (%)	98.26
			SD (%)	6.18

Table S6: AAV production titers of AAV variants generated in this study.

Typical genomic titers of AAV variants produced in this study. Titers are normalized to production in one 15 cm cell culture dish of HEK-293T cells, purification via PEG precipitation and resuspension in 0.5 ml 1x PBS.

AAV	Normalized production Titer (vg/ml)
SpyT587-AAV (mScarlet)	$5.1*10^{10}$
SpyT453-AAV (mScarlet)	$2.2*10^{10}$
SpyT587VP2-AAV (mScarlet)	$2.1*10^{10}$
SpyT453VP2-AAV (mScarlet)	$2.9*10^{10}$
SpyT453-AAV (linamarase-mScarlet)	$9.4*10^9$
wtAAV2 (mScarlet)	$6.0*10^{10}$

Table S7: AAV composition.

Name	Description	Plasmids
SpyT587-AAV (mScarlet)	pRC2(R585/588A)(587-SpyTag) AAV	AdH<br>pMH321<br>CMV-mScarlet
SpyT453-AAV (mScarlet)	pRC2(R585/588A)(453-SpyTag) AAV	AdH<br>pHJW414<br>CMV-mScarlet
SpyT587VP2-AAV (mScarlet)	pR2-VP1/3(R585/588A) + VP2(587-SpyTag)	AdH<br>pHJW162<br>pHJW341<br>CMV-mScarlet
SpyT453VP2-AAV (mScarlet)	pR2-VP1/3(R585/588A) + VP2(587-SpyTag)	AdH<br>pHJW162<br>pHJW351<br>CMV-mScarlet
SpyT453-AAV (linamarase-mScarlet)	pRC2(R585/588A)(453-SpyTag) AAV	AdH<br>pHJW414<br>pHJW427
AAV2 (mScarlet)	pRC2 AAV	AdH<br>pRC2<br>CMV-mScarlet

Table S8: Plasmids used and generated in this study.

Category	Plasmid Name	Description	Backbone/ Reference
AAV plasmids	AdH	Promoter-E2A-E4-VA-RNA	pHelper vector, Cell Biolabs (Cat.No. VPK-402, Part No. 340202)
	pRC2	pRC2 (AAV2 Rep-Cap vector)	pAAV-RC2, Cell Biolabs (Cat.No. VPK-402, Part No. VPK-422)
	pMH321	pRC2-587(SpyTag)-R585/588A	pAAV-RC2, Cell Biolabs (Cat.No. VPK-402, Part No. VPK-422)
	pHJW414	pRC2-453(SpyTag)-R585/588A	pAAV-RC2, Cell Biolabs (Cat.No. VPK-402, Part No. VPK-422)
	pHJW162	pR2-VP1/3(R585/588A)	pVP1/3 *(1)*
	pHJW341	pCMV-VP2-587(SpyTag)-R585/588A-VP3KO	pEGFP-C3 (Clontech)
	pHJW351	pCMV-VP2-453(SpyTag)-R585/588A-VP3KO	pEGFP-C3 (Clontech)
	CMV-mScarlet	ITR-pCMV-mScarlet-ITR	pCMV-mScarlet (D. Grimm)
	pHJW427	ITR-pCMV-Linamarase-2A-mScarlet-ITR	pCMV-mScarlet (D. Grimm), Linamarase: pWW315 *(2)*
SpyCatcher plasmids	pHJW410	pT7-SpyCatcher001Δ-GSS-Linker-DARPin_E01-His_6	pRSET, SpyCatcher-Toolbox1664, pHJW156 *(3)*
	pHJW415	pT7-SpyCatcher001Δ-GSS-Linker-DARPin_Ec1-His_6	pRSET, SpyCatcher-Toolbox1664, pMH327 *(3)*
	pHJW416	pT7-SpyCatcher001Δ-GSS-Linker-DARPin_9.29-His_6	pRSET, SpyCatcher-Toolbox1664, pMH328 *(3)*
	pHJW417	pT7-SpyCatcher001Δ-GSS-Linker-DARPin_D55.2-His_6	pRSET, SpyCatcher-Toolbox1664, pMH329 *(3)*
	pHJW422	pT7-SpyCatcher003-GSS-Linker-DARPin_E01-His_6	pRSET, pOSY115 *(3)*
	pHJW423	pT7-SpyCatcher001-GSS-Linker-DARPin_E01-His_6	pRSET, SpyCatcher-Toolbox1664
	pHJW424	pT7-SpyCatcher003Δ-GSS-Linker-DARPin_E01-His_6	pRSET, pOSY115 *(3)*

Table S9: Nucleic acid sequences of plasmids generated in this study.

Plasmid	Nucleic acid sequence (5' → 3')
**pMH321**<br><br>AAV2-Rep<br>AAV2-Cap<br>SpyTag001<br>R585A/R588A<br><br>Backbone:<br>pAAV-RC2,<br>(Cell Biolabs)	CGCAGCCGCCATGCCGGGGTTTTACGAGATTGTGATTAAGGTCCCCAGCGACCTT<br>GACGAGCATCTGCCCGGCATTTCTGACAGCTTTGTGAACTGGGTGGCCGAGAAGG<br>AATGGGAGTTGCCGCCAGATTCTGACATGGATCTGAATCTGATTGAGCAGGCACC<br>CCTGACCGTGGCCGAGAAGCTGCAGCGCGACTTTCTGACGGAATGGCGCCGTGTG<br>AGTAAGGCCCCGGAGGCCCTTTTCTTTGTGCAATTTGAGAAGGGAGAGAGCTACT<br>TCCACATGCACGTGCTCGTGGAAACCACCGGGGTGAAATCCATGGTTTTGGGACG<br>TTTCCTGAGTCAGATTCGCGAAAAACTGATTCAGAGAATTTACCGCGGGATCGAG<br>CCGACTTTGCCAAACTGGTTCGCGGTCACAAAGACCAGAAATGGCGCCGGAGGCG<br>GGAACAAGGTGGTGGATGAGTGCTACATCCCCAATTACTTGCTCCCCAAAACCCA<br>GCCTGAGCTCCAGTGGGCGTGGACTAATATGGAACAGTATTTAAGCGCCTGTTTG<br>AATCTCACGGAGCGTAAACGGTTGGTGGCGCAGCATCTGACGCACGTGTCGCAGA<br>CGCAGGAGCAGAACAAAGAGAATCAGAATCCCAATTCTGATGCGCCGGTGATCAG<br>ATCAAAAACTTCAGCCAGGTACATGGAGCTGGTCGGGTGGCTCGTGGACAAGGGG<br>ATTACCTCGGAGAAGCAGTGGATCCAGGAGGACCAGGCCTCATACATCTCCTTCA<br>ATGCGGCCTCCAACTCGCGGTCCCAAATCAAGGCTGCCTTGGACAATGCGGGAAA<br>GATTATGAGCCTGACTAAAACCGCCCCCGACTACCTGGTGGGCCAGCAGCCCGTG<br>GAGGACATTTCCAGCAATCGGATTTATAAAATTTGGAACTAAACGGGTACGATC<br>CCCAATATGCGGCTTCCGTCTTTCTGGGATGGGCCACGAAAAAGTTCGGCAAGAG<br>GAACACCATCTGGCTGTTTGGGCCTGCAACTACCGGGAAGACCAACATCGCGGAG<br>GCCATAGCCCACACTGTGCCCTTCTACGGGTGCGTAAACTGGACCAATGAGAACT<br>TTCCCTTCAACGACTGTGTCGACAAGATGGTGATCTGGTGGGAGGAGGGGAAGAT<br>GACCGCCAAGGTCGTGGAGTCGGCCAAAGCCATTCTCGGAGGAAGCAAGGTGCGC<br>GTGGACCAGAAATGCAAGTCCTCGGCCCAGATAGACCCGACTCCCGTGATCGTCA<br>CCTCCAACACCAACATGTGCGCCGTGATTGACGGGAACTCAACGACCTTCGAACA<br>CCAGCAGCCGTTGCAAGACCGGATGTTCAAATTTGAACTCACCCGCCGTCTGGAT<br>CATGACTTTGGGAAGGTCACCAAGCAGGAAGTCAAAGACTTTTTCCGGTGGGCAA<br>AGGATCACGTGGTTGAGGTGGAGCATGAATTCTACGTCAAAAAGGGTGGAGCCAA<br>GAAAAGACCCGCCCCCAGTGACGCAGATATAAGTGAGCCCAAACGGGTGCGCGAG<br>TCAGTTGCGCAGCCATCGACGTCAGACGCGGAAGCTTCGATCAACTACGCAGACA<br>GGTACCAAAACAAATGTTCTCGTCACGTGGGCATGAATCTGATGCTGTTTCCCTG<br>CAGACAATGCGAGAGAATGAATCAGAATTCAAATATCTGCTTCACTCACGGACAG<br>AAAGACTGTTTAGAGTGCTTTCCCGTGTCAGAATCTCAACCCGTTTCTGTCGTCA<br>AAAAGGCGTATCAGAAACTGTGCTACATTCATCATATCATGGGAAAGGTGCCAGA<br>CGCTTGCACTGCCTGCGATCTGGTCAATGTGGATTTGGATGACTGCATCTTTGAA<br>CAATAAATGATTTAAATCAGGTATGGCTGCCGATGGTTATCTTCCAGATTGGCTC<br>GAGGACACTCTCTCTGAAGGAATAAGACAGTGGTGGAAGCTCAAACCTGGCCCAC<br>CACCACCAAAGCCCGCAGAGCGGCATAAGGACGACAGCAGGGGTCTTGTGCTTCC<br>TGGGTACAAGTACCTCGGACCCTTCAACGGACTCGACAAGGGAGAGCCGGTCAAC<br>GAGGCAGACGCCGCGGCCCTCGAGCACGACAAAGCCTACGACCGGCAGCTCGACA<br>GCGGAGACAACCCGTACCTCAAGTACAACCACGCCGACGCGGAGTTTCAGGAGCG<br>CCTTAAAGAAGATACGTCTTTTGGGGGCAACCTCGGACGAGCAGTCTTCCAGGCG<br>AAAAAGAGGGTTCTTGAACCTCTGGGCCTGGTTGAGGAACCTGTTAAGACGGCTC<br>CGGGAAAAAAGAGGCCGGTAGAGCACTCTCCTGTGGAGCCAGACTCCTCCTCGGG<br>AACCGGAAAGGCGGGCCAGCAGCCTGCAAGAAAAAGATTGAATTTTGGTCAGACT<br>GGAGACGCAGACTCAGTACCTGACCCCCAGCCTCTCGGACAGCCACCAGCAGCCC<br>CCTCTGGTCTGGGAACTAATACGATGGCTACAGGCAGTGGCGCACCAATGGCAGA<br>CAATAACGAGGGCGCCGACGGAGTGGGTAATTCCTCGGGAAATTGGCATTGCGAT<br>TCCACATGGATGGGCGACAGAGTCATCACCACCAGCACCCGAACCTGGGCCCTGC<br>CCACCTACAACAACCACCTCTACAAACAAATTTCCAGCCAATCAGGAGCCTCGAA<br>CGACAATCACTACTTTGGCTACAGCACCCCTTGGGGGTATTTTGACTTCAACAGA<br>TTCCACTGCCACTTTTCACCACGTGACTGGCAAAGACTCATCAACAACAACTGGG

GATTCCGACCCAAGAGACTCAACTTCAAGCTCTTTAACATTCAAGTCAAAGAGGT
CACGCAGAATGACGGTACGACGACGATTGCCAATAACCTTACCAGCACGGTTCAG
GTGTTTACTGACTCGGAGTACCAGCTCCCGTACGTCCTCGGCTCGGCGCATCAAG
GATGCCTCCCGCCGTTCCCAGCAGACGTCTTCATGGTGCCACAGTATGGATACCT
CACCCTGAACAACGGGAGTCAGGCAGTAGGACGCTCTTCATTTTACTGCCTGGAG
TACTTTCCTTCTCAGATGCTGCGTACCGGAAACAACTTTACCTTCAGCTACACTT
TTGAGGACGTTCCTTTCCACAGCAGCTACGCTCACAGCCAGAGTCTGGACCGTCT
CATGAATCCTCTCATCGACCAGTACCTGTATTACTTGAGCAGAACAAACACTCCA
AGTGGAACCACCACGCAGTCAAGGCTTCAGTTTTCTCAGGCCGGAGCGAGTGACA
TTCGGGACCAGTCTAGGAACTGGCTTCCTGGACCCTGTTACCGCCAGCAGCGAGT
ATCAAAGACATCTGCGGATAACAACAACAGTGAATACTCGTGGACTGGAGCTACC
AAGTACCACCTCAATGGCAGAGACTCTCTGGTGAATCCGGGCCCGGCCATGGCAA
GCCACAAGGACGATGAAGAAAAGTTTTTTCCTCAGAGCGGGGTTCTCATCTTTGG
GAAGCAAGGCTCAGAGAAAACAAATGTGGACATTGAAAAGGTCATGATTACAGAC
GAAGAGGAAATCAGGACAACCAATCCCGTGGCTACGGAGCAGTATGGTTCTGTAT
CTACCAACCTCCAGGCAGGCAACGCCCACATCGTGATGGTGGACGCCTACAAGCC
CACCAAGGCACAAGCAGCTACCGCAGATGTCAACACACAAGGCGTTCTTCCAGGC
ATGGTCTGGCAGGACAGAGATGTGTACCTTCAGGGGCCCATCTGGGCAAAGATTC
CACACACGGACGGACATTTTCACCCCTCTCCCCTCATGGGTGGATTCGGACTTAA
ACACCCTCCTCCACAGATTCTCATCAAGAACACCCCGGTACCTGCGAATCCTTCG
ACCACCTTCAGTGCGGCAAAGTTTGCTTCCTTCATCACACAGTACTCCACGGGAC
AGGTCAGCGTGGAGATCGAGTGGGAGCTGCAGAAGGAAAACAGCAAACGCTGGAA
TCCCGAAATTCAGTACACTTCCAACTACAACAAGTCTGTTAATGTGGACTTTACT
GTGGACACTAATGGCGTGTATTCAGAGCCTCGCCCCATTGGCACCAGATACCTGA
CTCGTAATCTGTAATTGCTTGTTA

Plasmid	Nucleic acid sequence (5’ → 3’)
**pHJW414**	CGCAGCCGCCATGCCGGGGTTTTACGAGATTGTGATTAAGGTCCCCAGCGACCTT
	GACGAGCATCTGCCCGGCATTTCTGACAGCTTTGTGAACTGGGTGGCCGAGAAGG
	AATGGGAGTTGCCGCCAGATTCTGACATGGATCTGAATCTGATTGAGCAGGCACC
AAV2-Rep	CCTGACCGTGGCCGAGAAGCTGCAGCGCGACTTTCTGACGGAATGGCGCCGTGTG
AAV2-Cap	AGTAAGGCCCCGGAGGCCCTTTTCTTTGTGCAATTTGAGAAGGGAGAGAGCTACT
SpyTag001	TCCACATGCACGTGCTCGTGGAAACCACCGGGGTGAAATCCATGGTTTTGGGACG
R585A/R588A	TTTCCTGAGTCAGATTCGCGAAAAACTGATTCAGAGAATTTACCGCGGGATCGAG
	CCGACTTTGCCAAACTGGTTCGCGGTCACAAAGACCAGAAATGGCGCCGGAGGCG
	GGAACAAGGTGGTGGATGAGTGCTACATCCCCAATTACTTGCTCCCCAAAACCCA
Backbone:	GCCTGAGCTCCAGTGGGCGTGGACTAATATGGAACAGTATTTAAGCGCCTGTTTG
pAAV-RC2,	AATCTCACGGAGCGTAAACGGTTGGTGGCGCAGCATCTGACGCACGTGTCGCAGA
(Cell Biolabs)	CGCAGGAGCAGAACAAAGAGAATCAGAATCCCAATTCTGATGCGCCGGTGATCAG
	ATCAAAAACTTCAGCCAGGTACATGGAGCTGGTCGGGTGGCTCGTGGACAAGGGG
	ATTACCTCGGAGAAGCAGTGGATCCAGGAGGACCAGGCCTCATACATCTCCTTCA
	ATGCGGCCTCCAACTCGCGGTCCCAAATCAAGGCTGCCTTGGACAATGCGGGAAA
	GATTATGAGCCTGACTAAAACCGCCCCCGACTACCTGGTGGGCCAGCAGCCCGTG
	GAGGACATTTCCAGCAATCGGATTTATAAAATTTTGGAACTAAACGGGTACGATC
	CCCAATATGCGGCTTCCGTCTTTCTGGGATGGGCCACGAAAAAGTTCGGCAAGAG
	GAACACCATCTGGCTGTTTGGGCCTGCAACTACCGGGAAGACCAACATCGCGGAG
	GCCATAGCCCACACTGTGCCCTTCTACGGGTGCGTAAACTGGACCAATGAGAACT
	TTCCCTTCAACGACTGTGTCGACAAGATGGTGATCTGGTGGGAGGAGGGGAAGAT
	GACCGCCAAGGTCGTGGAGTCGGCCAAAGCCATTCTCGGAGGAAGCAAGGTGCGC
	GTGGACCAGAAATGCAAGTCCTCGGCCCAGATAGACCCGACTCCCGTGATCGTCA
	CCTCCAACACCAACATGTGCGCCGTGATTGACGGGAACTCAACGACCTTCGAACA
	CCAGCAGCCGTTGCAAGACCGGATGTTCAAATTTGAACTCACCCGCCGTCTGGAT
	CATGACTTTGGGAAGGTCACCAAGCAGGAAGTCAAAGACTTTTTCCGGTGGGCAA
	AGGATCACGTGGTTGAGGTGGAGCATGAATTCTACGTCAAAAAGGGTGGAGCCAA
	GAAAAGACCCGCCCCCAGTGACGCAGATATAAGTGAGCCCAAACGGGTGCGCGAG
	TCAGTTGCGCAGCCATCGACGTCAGACGCGGAAGCTTCGATCAACTACGCAGACA
	GGTACCAAAACAAATGTTCTCGTCACGTGGGCATGAATCTGATGCTGTTTCCCTG
	CAGACAATGCGAGAGAATGAATCAGAATTCAAATATCTGCTTCACTCACGGACAG
	AAAGACTGTTTAGAGTGCTTTCCCGTGTCAGAATCTCAACCCGTTTCTGTCGTCA
	AAAAGGCGTATCAGAAACTGTGCTACATTCATCATATCATGGGAAAGGTGCCAGA
	CGCTTGCACTGCCTGCGATCTGGTCAATGTGGATTTGGATGACTGCATCTTTGAA
	CAATAAATGATTTAAATCAGGTATGGCTGCCGATGGTTATCTTCCAGATTGGCTC
	GAGGACACTCTCTCTGAAGGAATAAGACAGTGGTGGAAGCTCAAACCTGGCCCAC
	CACCACCAAAGCCCGCAGAGCGGCATAAGGACGACAGCAGGGGTCTTGTGCTTCC
	TGGGTACAAGTACCTCGGACCCTTCAACGGACTCGACAAGGGAGAGCCGGTCAAC
	GAGGCAGACGCCGCGGCCCTCGAGCACGACAAAGCCTACGACCGGCAGCTCGACA
	GCGGAGACAACCCGTACCTCAAGTACAACCACGCCGACGCGGAGTTTCAGGAGCG
	CCTTAAAGAAGATACGTCTTTTGGGGGCAACCTCGGACGAGCAGTCTTCCAGGCG
	AAAAAGAGGGTTCTTGAACCTCTGGGCCTGGTTGAGGAACCTGTTAAGACGGCTC
	CGGGAAAAAAGAGGCCGGTAGAGCACTCTCCTGTGGAGCCAGACTCCTCCTCGGG
	AACCGGAAAGGCGGGCCAGCAGCCTGCAAGAAAAAGATTGAATTTTGGTCAGACT
	GGAGACGCAGACTCAGTACCTGACCCCCAGCCTCTCGGACAGCCACCAGCAGCCC
	CCTCTGGTCTGGGAACTAATACGATGGCTACAGGCAGTGGCGCACCAATGGCAGA
	CAATAACGAGGGCGCCGACGGAGTGGGTAATTCCTCGGGAAATTGGCATTGCGAT
	TCCACATGGATGGGCGACAGAGTCATCACCACCAGCACCCGAACCTGGGCCCTGC
	CCACCTACAACAACCACCTCTACAAACAAATTTCCAGCCAATCAGGAGCCTCGAA
	CGACAATCACTACTTTGGCTACAGCACCCCTTGGGGGTATTTTGACTTCAACAGA
	TTCCACTGCCACTTTTCACCACGTGACTGGCAAAGACTCATCAACAACAACTGGG
	GATTCCGACCCAAGAGACTCAACTTCAAGCTCTTTAACATTCAAGTCAAAGAGGT
	CACGCAGAATGACGGTACGACGATTGCCAATAACCTTACCAGCACGGTTCAG

GTGTTTACTGACTCGGAGTACCAGCTCCCGTACGTCCTCGGCTCGGCGCATCAAG
GATGCCTCCCGCCGTTCCCAGCAGACGTCTTCATGGTGCCACAGTATGGATACCT
CACCCTGAACAACGGGAGTCAGGCAGTAGGACGCTCTTCATTTTACTGCCTGGAG
TACTTTCCTTCTCAGATGCTGCGTACCGGAAACAACTTTACCTTCAGCTACACTT
TTGAGGACGTTCCTTTCCACAGCAGCTACGCTCACAGCCAGAGTCTGGACCGTCT
CATGAATCCTCTCATCGACCAGTACCTGTATTACTTGAGCAGAACAAACACTCCA
AGTGGAGCCCACATCGTGATGGTGGACGCCTACAAGCCCACCAAGACCACCACGC
AGTCAAGGCTTCAGTTTTCTCAGGCCGGAGCGAGTGACATTCGGGACCAGTCTAG
GAACTGGCTTCCTGGACCCTGTTACCGCCAGCAGCGAGTATCAAAGACATCTGCG
GATAACAACAACAGTGAATACTCGTGGACTGGAGCTACCAAGTACCACCTCAATG
GCAGAGACTCTCTGGTGAATCCGGGCCCGGCCATGGCAAGCCACAAGGACGATGA
AGAAAAGTTTTTTCCTCAGAGCGGGGTTCTCATCTTTGGGAAGCAAGGCTCAGAG
AAAACAAATGTGGACATTGAAAAGGTCATGATTACAGACGAAGAGGAAATCAGGA
CAACCAATCCCGTGGCTACGGAGCAGTATGGTTCTGTATCTACCAACCTCCAGGC
AGGCAACGCACAAGCAGCTACCGCAGATGTCAACACACAAGGCGTTCTTCCAGGC
ATGGTCTGGCAGGACAGAGATGTGTACCTTCAGGGGCCCATCTGGGCAAAGATTC
CACACACGGACGGACATTTTCACCCCTCTCCCCTCATGGGTGGATTCGGACTTAA
ACACCCTCCTCCACAGATTCTCATCAAGAACACCCCGGTACCTGCGAATCCTTCG
ACCACCTTCAGTGCGGCAAAGTTTGCTTCCTTCATCACACAGTACTCCACGGGAC
AGGTCAGCGTGGAGATCGAGTGGGAGCTGCAGAAGGAAAACAGCAAACGCTGGAA
TCCCGAAATTCAGTACACTTCCAACTACAACAAGTCTGTTAATGTGGACTTTACT
GTGGACACTAATGGCGTGTATTCAGAGCCTCGCCCCATTGGCACCAGATACCTGA
CTCGTAATCTGTAATTGCTTGTTA

Plasmid	Nucleic acid sequence (5’ → 3’)
**pHJW162**<br><br>AAV2-Rep<br>AAV2-Cap<br>Mutated VP2<br>start codon<br>(ACG >> GCG)<br>R585A/R588A<br><br><u>Backbone:</u><br>pAAV-RC2,<br>(Cell Biolabs)	CGCAGCCGCCATGCCGGGGTTTTACGAGATTGTGATTAAGGTCCCCAGCGACCTT<br>GACGAGCATCTGCCCGGCATTTCTGACAGCTTTGTGAACTGGGTGGCCGAGAAGG<br>AATGGGAGTTGCCGCCAGATTCTGACATGGATCTGAATCTGATTGAGCAGGCACC<br>CCTGACCGTGGCCGAGAAGCTGCAGCGCGACTTTCTGACGGAATGGCGCCGTGTG<br>AGTAAGGCCCCGGAGGCCCTTTTCTTTGTGCAATTTGAGAAGGGAGAGAGCTACT<br>TCCACATGCACGTGCTCGTGGAAACCACCGGGGTGAAATCCATGGTTTTGGGACG<br>TTTCCTGAGTCAGATTCGCGAAAAACTGATTCAGAGAATTTACCGCGGGATCGAG<br>CCGACTTTGCCAAACTGGTTCGCGGTCACAAAGACCAGAAATGGCGCCGGAGGCG<br>GGAACAAGGTGGTGGATGAGTGCTACATCCCCAATTACTTGCTCCCCAAAACCCA<br>GCCTGAGCTCCAGTGGGCGTGGACTAATATGGAACAGTATTTAAGCGCCTGTTTG<br>AATCTCACGGAGCGTAAACGGTTGGTGGCGCAGCATCTGACGCACGTGTCGCAGA<br>CGCAGGAGCAGAACAAAGAGAATCAGAATCCCAATTCTGATGCGCCGGTGATCAG<br>ATCAAAAACTTCAGCCAGGTACATGGAGCTGGTCGGGTGGCTCGTGGACAAGGGG<br>ATTACCTCGGAGAAGCAGTGGATCCAGGAGGACCAGGCCTCATACATCTCCTTCA<br>ATGCGGCCTCCAACTCGCGGTCCCAAATCAAGGCTGCCTTGGACAATGCGGGAAA<br>GATTATGAGCCTGACTAAAACCGCCCCCGACTACCTGGTGGGCCAGCAGCCCGTG<br>GAGGACATTTCCAGCAATCGGATTTATAAAATTTTGGAACTAAACGGGTACGATC<br>CCCAATATGCGGCTTCCGTCTTTCTGGGATGGGCCACGAAAAAGTTCGGCAAGAG<br>GAACACCATCTGGCTGTTTGGGCCTGCAACTACCGGGAAGACCAACATCGCGGAG<br>GCCATAGCCCACACTGTGCCCTTCTACGGGTGCGTAAACTGGACCAATGAGAACT<br>TTCCCTTCAACGACTGTGTCGACAAGATGGTGATCTGGTGGGAGGAGGGGAAGAT<br>GACCGCCAAGGTCGTGGAGTCGGCCAAAGCCATTCTCGGAGGAAGCAAGGTGCGC<br>GTGGACCAGAAATGCAAGTCCTCGGCCCAGATAGACCCGACTCCCGTGATCGTCA<br>CCTCCAACACCAACATGTGCGCCGTGATTGACGGGAACTCAACGACCTTCGAACA<br>CCAGCAGCCGTTGCAAGACCGGATGTTCAAATTTGAACTCACCCGCCGTCTGGAT<br>CATGACTTTGGGAAGGTCACCAAGCAGGAAGTCAAAGACTTTTTCCGGTGGGCAA<br>AGGATCACGTGGTTGAGGTGGAGCATGAATTCTACGTCAAAAAGGGTGGAGCCAA<br>GAAAAGACCCGCCCCCAGTGACGCAGATATAAGTGAGCCCAAACGGGTGCGCGAG<br>TCAGTTGCGCAGCCATCGACGTCAGACGCGGAAGCTTCGATCAACTACGCAGACA<br>GGTACCAAAACAAATGTTCTCGTCACGTGGGCATGAATCTGATGCTGTTTCCCTG<br>CAGACAATGCGAGAGAATGAATCAGAATTCAAATATCTGCTTCACTCACGGACAG<br>AAAGACTGTTTAGAGTGCTTTCCCGTGTCAGAATCTCAACCCGTTTCTGTCGTCA<br>AAAAGGCGTATCAGAAACTGTGCTACATTCATCATATCATGGGAAAGGTGCCAGA<br>CGCTTGCACTGCCTGCGATCTGGTCAATGTGGATTTGGATGACTGCATCTTTGAA<br>CAATAAATGATTTAAATCAGGTATGGCTGCCGATGGTTATCTTCCAGATTGGCTC<br>GAGGACACTCTCTCTGAAGGAATAAGACAGTGGTGGAAGCTCAAACCTGGCCCAC<br>CACCACCAAAGCCCGCAGAGCGGCATAAGGACGACAGCAGGGGTCTTGTGCTTCC<br>TGGGTACAAGTACCTCGGACCCTTCAACGGACTCGACAAGGGAGAGCCGGTCAAC<br>GAGGCAGACGCCGCGGCCCTCGAGCACGACAAAGCCTACGACCGGCAGCTCGACA<br>GCGGAGACAACCCGTACCTCAAGTACAACCACGCCGACGCGGAGTTTCAGGAGCG<br>CCTTAAAGAAGATACGTCTTTTGGGGGCAACCTCGGACGAGCAGTCTTCCAGGCG<br>AAAAAGAGGGTTCTTGAACCTCTGGGCCTGGTTGAGGAACCTGTTAAGGCGGCTC<br>CGGGAAAAAAGAGGCCGGTAGAGCACTCTCCTGTGGAGCCAGACTCCTCCTCGGG<br>AACCGGAAAGGCGGGCCAGCAGCCTGCAAGAAAAAGATTGAATTTTGGTCAGACT<br>GGAGACGCAGACTCAGTACCTGACCCCCAGCCTCTCGGACAGCCACCAGCAGCCC<br>CCTCTGGTCTGGGAACTAATACGATGGCTACAGGCAGTGGCGCACCAATGGCAGA<br>CAATAACGAGGGCGCCGACGGAGTGGGTAATTCCTCGGGAAATTGGCATTGCGAT<br>TCCACATGGATGGGCGACAGAGTCATCACCACCAGCACCCGAACCTGGGCCCTGC<br>CCACCTACAACAACCACCTCTACAAACAAATTTCCAGCCAATCAGGAGCCTCGAA<br>CGACAATCACTACTTTGGCTACAGCACCCCTTGGGGGTATTTTGACTTCAACAGA<br>TTCCACTGCCACTTTTCACCACGTGACTGGCAAAGACTCATCAACAACAACTGGG<br>GATTCCGACCCAAGAGACTCAACTTCAAGCTCTTTAACATTCAAGTCAAAGAGGT<br>CACGCAGAATGACGGTACGACGACGATTGCCAATAACCTTACCAGCACGGTTCAG

GTGTTTACTGACTCGGAGTACCAGCTCCCGTACGTCCTCGGCTCGGCGCATCAAG
GATGCCTCCCGCCGTTCCCAGCAGACGTCTTCATGGTGCCACAGTATGGATACCT
CACCCTGAACAACGGGAGTCAGGCAGTAGGACGCTCTTCATTTTACTGCCTGGAG
TACTTTCCTTCTCAGATGCTGCGTACCGGAAACAACTTTACCTTCAGCTACACTT
TTGAGGACGTTCCTTTCCACAGCAGCTACGCTCACAGCCAGAGTCTGGACCGTCT
CATGAATCCTCTCATCGACCAGTACCTGTATTACTTGAGCAGAACAAACACTCCA
AGTGGAACCACCACGCAGTCAAGGCTTCAGTTTTCTCAGGCCGGAGCGAGTGACA
TTCGGGACCAGTCTAGGAACTGGCTTCCTGGACCCTGTTACCGCCAGCAGCGAGT
ATCAAAGACATCTGCGGATAACAACAACAGTGAATACTCGTGGACTGGAGCTACC
AAGTACCACCTCAATGGCAGAGACTCTCTGGTGAATCCGGGCCCGGCCATGGCAA
GCCACAAGGACGATGAAGAAAAGTTTTTTCCTCAGAGCGGGGTTCTCATCTTTGG
GAAGCAAGGCTCAGAGAAAACAAATGTGGACATTGAAAAGGTCATGATTACAGAC
GAAGAGGAAATCAGGACAACCAATCCCGTGGCTACGGAGCAGTATGGTTCTGTAT
CTACCAACCTCCAGGCAGGCAACGCACAAGCAGCTACCGCAGATGTCAACACACA
AGGCGTTCTTCCAGGCATGGTCTGGCAGGACAGAGATGTGTACCTTCAGGGGCCC
ATCTGGGCAAAGATTCCACACACGGACGGACATTTTCACCCCTCTCCCCTCATGG
GTGGATTCGGACTTAAACACCCTCCTCCACAGATTCTCATCAAGAACACCCCGGT
ACCTGCGAATCCTTCGACCACCTTCAGTGCGGCAAAGTTTGCTTCCTTCATCACA
CAGTACTCCACGGGACAGGTCAGCGTGGAGATCGAGTGGGAGCTGCAGAAGGAAA
ACAGCAAACGCTGGAATCCCGAAATTCAGTACACTTCCAACTACAACAAGTCTGT
TAATGTGGACTTTACTGTGGACACTAATGGCGTGTATTCAGAGCCTCGCCCCATT
GGCACCAGATACCTGACTCGTAATCTGTAATTGCTTGTTA

Plasmid	Nucleic acid sequence (5’ → 3’)
**pHJW341**	CGCCATGCATTAGTTATTAATAGTAATCAATTACGGGGTCATTAGTTCATAGCCC
	ATATATGGAGTTCCGCGTTACATAACTTACGGTAAATGGCCCGCCTGGCTGACCG
	CCCAACGACCCCCGCCCATTGACGTCAATAATGACGTATGTTCCCATAGTAACGC
CMV promoter	CAATAGGGACTTTCCATTGACGTCAATGGGTGGAGTATTTACGGTAAACTGCCCA
AAV2-Cap	CTTGGCAGTACATCAAGTGTATCATATGCCAAGTACGCCCCCTATTGACGTCAAT
Mutated VP3	GACGGTAAATGGCCCGCCTGGCATTATGCCCAGTACATGACCTTATGGGACTTTC
start codon	CTACTTGGCAGTACATCTACGTATTAGTCATCGCTATTACCATGGTGATGCGGTT
(ATG >> ATC)	TTGGCAGTACATCAATGGGCGTGGATAGCGGTTTGACTCACGGGGATTTCCAAGT
SpyTag001	CTCCACCCCATTGACGTCAATGGGAGTTTGTTTTGGCACCAAAATCAACGGGACT
R585A/R588A	TTCCAAAATGTCGTAACAACTCCGCCCCATTGACGCAAATGGGCGGTAGGCGTGT
PolyA	ACGGTGGGAGGTCTATATAAGCAGAGCTGGTTTAGTGAACCGTCAGATCCGCTAG
	CGCTACCGGTCGCCACCATGCCGGGAAAAAAGAGGCCGGTAGAGCACTCTCCTGT
Backbone:	GGAGCCAGACTCCTCCTCGGGAACCGGAAAGGCGGGCCAGCAGCCTGCAAGAAAA
pEGFP-C3	AGATTGAATTTTGGTCAGACTGGAGACGCAGACTCAGTACCTGACCCCCAGCCTC
(Clontech)	TCGGACAGCCACCAGCAGCCCCCTCTGGTCTGGGAACTAATACGATCGCTACAGG
	CAGTGGCGCACCAATGGCAGACAATAACGAGGGCGCCGACGGAGTGGGTAATTCC
	TCGGGAAATTGGCATTGCGATTCCACATGGATGGGCGACAGAGTCATCACCACCA
	GCACCCGAACCTGGGCCCTGCCCACCTACAACAACCACCTCTACAAACAAATTTC
	CAGCCAATCAGGAGCCTCGAACGACAATCACTACTTTGGCTACAGCACCCCTTGG
	GGGTATTTTGACTTCAACAGATTCCACTGCCACTTTTCACCACGTGACTGGCAAA
	GACTCATCAACAACAACTGGGGATTCCGACCCAAGAGACTCAACTTCAAGCTCTT
	TAACATTCAAGTCAAAGAGGTCACGCAGAATGACGGTACGACGACGATTGCCAAT
	AACCTTACCAGCACGGTTCAGGTGTTTACTGACTCGGAGTACCAGCTCCCGTACG
	TCCTCGGCTCGGCGCATCAAGGATGCCTCCCGCCGTTCCCAGCAGACGTCTTCAT
	GGTGCCACAGTATGGATACCTCACCCTGAACAACGGGAGTCAGGCAGTAGGACGC
	TCTTCATTTTACTGCCTGGAGTACTTTCCTTCTCAGATGCTGCGTACCGGAAACA
	ACTTTACCTTCAGCTACACTTTTGAGGACGTTCCTTTCCACAGCAGCTACGCTCA
	CAGCCAGAGTCTGGACCGTCTCATGAATCCTCTCATCGACCAGTACCTGTATTAC
	TTGAGCAGAACAAACACTCCAAGTGGAACCACCACGCAGTCAAGGCTTCAGTTTT
	CTCAGGCCGGAGCGAGTGACATTCGGGACCAGTCTAGGAACTGGCTTCCTGGACC
	CTGTTACCGCCAGCAGCGAGTATCAAAGACATCTGCGGATAACAACAACAGTGAA
	TACTCGTGGACTGGAGCTACCAAGTACCACCTCAATGGCAGAGACTCTCTGGTGA
	ATCCGGGCCCGGCCATGGCAAGCCACAAGGACGATGAAGAAAAGTTTTTTCCTCA
	GAGCGGGGTTCTCATCTTTGGGAAGCAAGGCTCAGAGAAAACAAATGTGGACATT
	GAAAAGGTCATGATTACAGACGAAGAGGAAATCAGGACAACCAATCCCGTGGCTA
	CGGAGCAGTATGGTTCTGTATCTACCAACCTCCAGGCAGGCAACACCGGCGCCCA
	CATCGTGATGGTGGACGCCTACAAGCCCACCAAGGGCCTGAGCGCACAAGCAGCT
	ACCGCAGATGTCAACACACAAGGCGTTCTTCCAGGCATGGTCTGGCAGGACAGAG
	ATGTGTACCTTCAGGGGCCCATCTGGGCAAAGATTCCACACACGGACGGACATTT
	TCACCCCTCTCCCCTCATGGGTGGATTCGGACTTAAACACCCTCCTCCACAGATT
	CTCATCAAGAACACCCCGGTACCTGCGAATCCTTCGACCACCTTCAGTGCGGCAA
	AGTTTGCTTCCTTCATCACACAGTACTCCACGGGACAGGTCAGCGTGGAGATCGA
	GTGGGAGCTGCAGAAGGAAAACAGCAAACGCTGGAATCCCGAAATTCAGTACACT
	TCCAACTACAACAAGTCTGTTAATGTGGACTTTACTGTGGACACTAATGGCGTGT
	ATTCAGAGCCTCGCCCCATTGGCACCAGATACCTGACTCGTAATCTGTAAGATCT
	CGAGCTCAAGCTTCGAATTCTGCAGTCGACGGTACCGCGGGCCCGGGATCCACCG
	GATCTAGATAACTGATCATAATCAGCCATACCACATTTGTAGAGGTTTTACTTGC
	TTTAAAAAACCTCCCACACCTCCCCCTGAACCTGAAACATAAAATGAATGCAATT
	GTTGTTGTTAACTTGTTTATTGCAGCTTATAATGGTTACAAATAAAGCAATAGCA
	TCACAAATTTCACAAATAAAGCATTTTTTT

Plasmid	Nucleic acid sequence (5’ → 3’)
**pHJW351**<br><br>CMV promoter<br>AAV2-Cap<br>Mutated VP3<br>start codon<br>(ATG >> ATC)<br>SpyTag001<br>R585A/R588A<br>PolyA<br><br>Backbone:<br>pEGFP-C3<br>(Clontech)	CGCCATGCATTAGTTATTAATAGTAATCAATTACGGGGTCATTAGTTCATAGCCC<br>ATATATGGAGTTCCGCGTTACATAACTTACGGTAAATGGCCCGCCTGGCTGACCG<br>CCCAACGACCCCCGCCCATTGACGTCAATAATGACGTATGTTCCCATAGTAACGC<br>CAATAGGGACTTTCCATTGACGTCAATGGGTGGAGTATTTACGGTAAACTGCCCA<br>CTTGGCAGTACATCAAGTGTATCATATGCCAAGTACGCCCCCTATTGACGTCAAT<br>GACGGTAAATGGCCCGCCTGGCATTATGCCCAGTACATGACCTTATGGGACTTTC<br>CTACTTGGCAGTACATCTACGTATTAGTCATCGCTATTACCATGGTGATGCGGTT<br>TTGGCAGTACATCAATGGGCGTGGATAGCGGTTTGACTCACGGGGATTTCCAAGT<br>CTCCACCCCATTGACGTCAATGGGAGTTTGTTTTGGCACCAAAATCAACGGGACT<br>TTCCAAAATGTCGTAACAACTCCGCCCCATTGACGCAAATGGGCGGTAGGCGTGT<br>ACGGTGGGAGGTCTATATAAGCAGAGCTGGTTTAGTGAACCGTCAGATCCGCTAG<br>CGCTACCGGTCGCCACCATGCCGGGAAAAAAGAGGCCGGTAGAGCACTCTCCTGT<br>GGAGCCAGACTCCTCCTCGGGAACCGGAAAGGCGGGCCAGCAGCCTGCAAGAAAA<br>AGATTGAATTTTGGTCAGACTGGAGACGCAGACTCAGTACCTGACCCCCAGCCTC<br>TCGGACAGCCACCAGCAGCCCCCTCTGGTCTGGGAACTAATACGATCGCTACAGG<br>CAGTGGCGCACCAATGGCAGACAATAACGAGGGCGCCGACGGAGTGGGTAATTCC<br>TCGGGAAATTGGCATTGCGATTCCACATGGATGGGCGACAGAGTCATCACCACCA<br>GCACCCGAACCTGGGCCCTGCCCACCTACAACAACCACCTCTACAAACAAATTTC<br>CAGCCAATCAGGAGCCTCGAACGACAATCACTACTTTGGCTACAGCACCCCTTGG<br>GGGTATTTTGACTTCAACAGATTCCACTGCCACTTTTCACCACGTGACTGGCAAA<br>GACTCATCAACAACAACTGGGGATTCCGACCCAAGAGACTCAACTTCAAGCTCTT<br>TAACATTCAAGTCAAAGAGGTCACGCAGAATGACGGTACGACGACGATTGCCAAT<br>AACCTTACCAGCACGGTTCAGGTGTTTACTGACTCGGAGTACCAGCTCCCGTACG<br>TCCTCGGCTCGGCGCATCAAGGATGCCTCCCGCCGTTCCCAGCAGACGTCTTCAT<br>GGTGCCACAGTATGGATACCTCACCCTGAACAACGGGAGTCAGGCAGTAGGACGC<br>TCTTCATTTTACTGCCTGGAGTACTTTCCTTCTCAGATGCTGCGTACCGGAAACA<br>ACTTTACCTTCAGCTACACTTTTGAGGACGTTCCTTTCCACAGCAGCTACGCTCA<br>CAGCCAGAGTCTGGACCGTCTCATGAATCCTCTCATCGACCAGTACCTGTATTAC<br>TTGAGCAGAACAAACACTCCAAGTGGAGCCCACATCGTGATGGTGGACGCCTACA<br>AGCCCACCAAGACCACCACGCAGTCAAGGCTTCAGTTTTCTCAGGCCGGAGCGAG<br>TGACATTCGGGACCAGTCTAGGAACTGGCTTCCTGGACCCTGTTACCGCCAGCAG<br>CGAGTATCAAAGACATCTGCGGATAACAACAACAGTGAATACTCGTGGACTGGAG<br>CTACCAAGTACCACCTCAATGGCAGAGACTCTCTGGTGAATCCGGGCCCGGCCAT<br>GGCAAGCCACAAGGACGATGAAGAAAAGTTTTTCCTCAGAGCGGGGTTCTCATC<br>TTTGGGAAGCAAGGCTCAGAGAAAACAAATGTGGACATTGAAAAGGTCATGATTA<br>CAGACGAAGAGGAAATCAGGACAACCAATCCCGTGGCTACGGAGCAGTATGGTTC<br>TGTATCTACCAACCTCCAGGCAGGCAACGCACAAGCAGCTACCGCAGATGTCAAC<br>ACACAAGGCGTTCTTCCAGGCATGGTCTGGCAGGACAGAGATGTGTACCTTCAGG<br>GGCCCATCTGGGCAAAGATTCCACACACGGACGGACATTTTCACCCCTCTCCCCT<br>CATGGGTGGATTCGGACTTAAACACCCTCCTCCACAGATTCTCATCAAGAACACC<br>CCGGTACCTGCGAATCCTTCGACCACCTTCAGTGCGGCAAAGTTTGCTTCCTTCA<br>TCACACAGTACTCCACGGGACAGGTCAGCGTGGAGATCGAGTGGGAGCTGCAGAA<br>GGAAAACAGCAAACGCTGGAATCCCGAAATTCAGTACACTTCCAACTACAACAAG<br>TCTGTTAATGTGGACTTTACTGTGGACACTAATGGCGTGTATTCAGAGCCTCGCC<br>CCATTGGCACCAGATACCTGACTCGTAATCTGTAAGATCTCGAGCTCAAGCTTCG<br>AATTCTGCAGTCGACGGTACCGCGGGCCCGGGATCCACCGGATCTAGATAACTGA<br>TCATAATCAGCCATACCACATTTGTAGAGGTTTTACTTGCTTTAAAAAACCTCCC<br>ACACCTCCCCCTGAACCTGAAACATAAAATGAATGCAATTGTTGTTGTTAACTTG<br>TTTATTGCAGCTTATAATGGTTACAAATAAAGCAATAGCATCACAAATTTCACAA<br>ATAAAGCATTTTTTT

Plasmid	Nucleic acid sequence (5’ → 3’)
**CMV-mScarlet**<br><br>AAV2-ITR<br>CMV promoter<br>mScarlet<br>AAV4-ITR<br><br>Backbone:<br>pCMV-mScarlet<br>(D. Grimm)	GGGGGGGGGGCCACTCCCTCTCTGCGCGCTCGCTCGCTCACTGAGGCCGGGCGAC<br>CAAAGGTCGCCCGACGCCCGGGCTTTGCCCGGGCGGCCTCAGTGAGCGAGCGAGC<br>GCGCAGAGAGGGAGTGGCCAACTCCATCACTAGGGGTTCCTAGATCTGATATCGT<br>CGACGTCGAGGTTACATAACTTACGGTAAATGGCCCGCCTGGCTGACCGCCCAAC<br>GACCCCCGCCCATTGACGTCAATAATGACGTATGTTCCCATAGTAACGCCAATAG<br>GGACTTTCCATTGACGTCAATGGGTGGAGTATTTACGGTAAACTGCCCACTTGGC<br>AGTACATCAAGTGTATCATATGCCAAGTACGCCCCCTATTGACGTCAATGACGGT<br>AAATGGCCCGCCTGGCATTATGCCCAGTACATGACCTTATGGGACTTTCCTACTT<br>GGCAGTACATCTACGTATTAGTCATCGCTATTACCATGGTGATGCGGTTTTGGCA<br>GTACATCAATGGGCGTGGATAGCGGTTTGACTCACGGGGATTTCCAAGTCTCCAC<br>CCCATTGACGTCAATGGGAGTTTGTTTTGGCACCAAAATCAACGGGACTTTCCAA<br>AATGTCGTAACAACTCCGCCCCATTGACGCAAATGGGCGGTAGGCGTGTACGGTG<br>GGAGGTCTATATAAGCAGAGCTGGTTTAGTGAACCGTCAGATCCGGATCCACCGG<br>TGCCACCATGGTGAGCAAGGGCGAGGCAGTGATCAAGGAGTTCATGCGGTTCAAG<br>GTGCACATGGAGGGCTCCATGAACGGCCACGAGTTCGAGATCGAGGGCGAGGGCG<br>AGGGCCGCCCCTACGAGGGCACCCAGACCGCCAAGCTGAAGGTGACCAAGGGTGG<br>CCCCCTGCCCTTCTCCTGGGACATCCTGTCCCCTCAGTTCATGTACGGCTCCAGG<br>GCCTTCACCAAGCACCCCGCCGACATCCCCGACTACTATAAGCAGTCCTTCCCCG<br>AGGGCTTCAAGTGGGAGCGCGTGATGAACTTCGAGGACGGCGGCGCCGTGACCGT<br>GACCCAGGACACCTCCCTGGAGGACGGCACCCTGATCTACAAGGTGAAGCTCCGC<br>GGCACCAACTTCCCTCCTGACGGCCCCGTAATGCAGAAGAAGACAATGGGCTGGG<br>AAGCGTCCACCGAGCGGTTGTACCCCGAGGACGGCGTGCTGAAGGGCGACATTAA<br>GATGGCCCTGCGCCTGAAGGACGGCGGCCGCTACCTGGCGGACTTCAAGACCACC<br>TACAAGGCCAAGAAGCCCGTGCAGATGCCCGGCGCCTACAACGTCGACCGCAAGT<br>TGGACATCACCTCCCACAACGAGGACTACACCGTGGTGGAACAGTACGAACGCTC<br>CGAGGGCCGCCACTCCACCGGCGGCATGGACGAGCTGTACAAGTCCGGACTCAGA<br>TCTCGAGCTCAAGCTTCGAATTCTGCAGTCGACGGTACCGCGGGCCCGGGATCCA<br>CCGGATCTAGATAAGCTAGCAATAAAGGATCGTTTATTTTCATTGGAAGCGTGTG<br>TTGGTTTTTTGATCACTCGAGGCGGCCGCAAAAAAGTAACGACGCGACGACGTAA<br>CTCTTGATTACGTGGTCGCGTCGTTACGGTGTTTCGTCCTTTCCACAAGATATAT<br>AAAGCCAAGAAATCGAAATACTTTCAAGTTACGGTAAGCATATGATAGTCCATTT<br>TAAAACATAATTTTAAAACTGCAAACTACCCAAGAAATTATTACTTTCTACGTCA<br>CGTATTTTGTACTAATATCTTTGTGTTTACAGTCAAATTAATTCTAATTATCTCT<br>CTAACAGCCTTGTATCGTATATGCAAATATGAAGGAATCATGGGAAATAGGCCCT<br>CTTCCTGCCCGACCTTGGCGCGCGCTCGGCGCGCGGTCACGCTCCGTCACGTGGT<br>GCGTTTTGCCTGCGCGTCTTTCCACTGGGGCCGGGGCGGCGCGGAGGGTTCAGAC<br>GCGGGCTCTTACTCAAACGCTGATCCCCGAAACTACCGAGCAGTGTCTGCGCGAC<br>GCGAAAGCATTTTTGGAGCGAGGGGCCATGGTGCCCATGGGATTCCCACGGGTGT<br>TGGACTCGGGCGCGCCGATATCAGATCTGGGAAACCAGATGATGGAGGTACCCAC<br>TCCCTCTATGCGCGCTCGCTCACTCACTCGGCCCTGCCGGCCAGAGGCCGGCAGT<br>CTGGAGACCTTTGGTCTCCAGGGCCGAGTGAGTGAGCGAGCGCGCATAGAGGGAG<br>TGGGTAGGACGCG

Plasmid	Nucleic acid sequence (5’ → 3’)
**pHJW427**<br><br>AAV2-ITR<br>CMV promoter<br>Linamarase<br>mScarlet<br>AAV4-ITR<br><br>Backbone:<br>pCMV-<br>mScarlet<br>(D. Grimm)	GGGGGGGGGGCCACTCCCTCTCTGCGCGCTCGCTCGCTCACTGAGGCCGGGCGAC<br>CAAAGGTCGCCCGACGCCCGGGCTTTGCCCGGGCGGCCTCAGTGAGCGAGCGAGC<br>GCGCAGAGAGGGAGTGGCCAACTCCATCACTAGGGGTTCCTAGATCTGATATCGT<br>CGACGTCGAGGTTACATAACTTACGGTAAATGGCCCGCCTGGCTGACCGCCCAAC<br>GACCCCCGCCCATTGACGTCAATAATGACGTATGTTCCCATAGTAACGCCAATAG<br>GGACTTTCCATTGACGTCAATGGGTGGAGTATTTACGGTAAACTGCCCACTTGGC<br>AGTACATCAAGTGTATCATATGCCAAGTACGCCCCCTATTGACGTCAATGACGGT<br>AAATGGCCCGCCTGGCATTATGCCCAGTACATGACCTTATGGGACTTTCCTACTT<br>GGCAGTACATCTACGTATTAGTCATCGCTATTACCATGGTGATGCGGTTTTGGCA<br>GTACATCAATGGGCGTGGATAGCGGTTTGACTCACGGGGATTTCCAAGTCTCCAC<br>CCCATTGACGTCAATGGGAGTTTGTTTTGGCACCAAAATCAACGGGACTTTCCAA<br>AATGTCGTAACAACTCCGCCCCATTGACGCAAATGGGCGGTAGGCGTGTACGGTG<br>GGAGGTCTATATAAGCAGAGCTGGTTTAGTGAACCGTCAGATCCGGATCCACCGG<br>TGCCACCATGCTCGTCTTGTTCATAAGCTTGTTGGCTCTCACTAGGCCAGCAATG<br>GGAACTGATGATGATGATGATAATATTCCTGACGATTTTAGCCGTAAATATTTTC<br>CAGATGACTTCATTTTTGGAACGGCTACTTCTGCTTATCAGATCGAAGGTGAAGC<br>AACCGCAAAGGGTAGAGCACCTAGTGTTTGGGACATATTTTCCAAGGAGACTCCA<br>GATAGAATATTAGATGGCAGCAATGGAGACGTTGCAGTTGATTTCTATAACCGCT<br>ACATACAAGATATAAAAAACGTCAAAAAGATGGGTTTTAATGCATTTAGAATGTC<br>CATTTCATGGTCTAGAGTTATACCATCCGGAAGGAGACGTGAAGGAGTGAACGAG<br>GAAGGAATTCAATTCTACAATGATGTTATCAATGAAATTATAAGCAATGGACTAG<br>AGCCTTTTGTTACTATTTTTCATTGGGATACTCCTCAAGCACTGCAGGACAAATA<br>TGGTGGCTTCTTAAGCCGTGATATTGTGTACGATTATCTCCAATATGCAGATCTT<br>CTCTTTGAAAGATTCGGTGATCGAGTGAAACCGTGGATGACTTTTAATGAACCAT<br>CAGCATATGTTGGATTTGCCCATGATGATGGAGTTTTTGCCCCTGGTCGATGCTC<br>ATCTTGGGTGAATCGCCAATGCCTAGCTGGAGACTCAGCCACAGAACCTTATATA<br>GTTGCCCATAATTTGCTTCTTTCTCATGCTGCAGCTGTTCACCAATATAGAAAAT<br>ATTATCAGGGAACTCAAAAGGGCAAGATTGGGATTACCCTCTTTACCTTCTGGTA<br>TGAACCTCTCTCCGACAGTAAAGTTGATGTGCAAGCAGCCAAAACAGCCTTAGAT<br>TTCATGTTTGGATTGTGGATGGATCCCATGACTTATGGACGATATCCAAGAACTA<br>TGGTAGATTTAGCCGGAGATAAATTGATTGGATTTACAGATGAAGAATCTCAATT<br>ACTTAGGGGATCATATGATTTTGTTGGATTACAATACTACACTGCATATTATGCA<br>GAACCAATTCCTCCAGTTGATCCAAAATTTCGTAGATACAAAACTGATAGTGGTG<br>TTAATGCGACTCCTTACGATCTTAATGGTAATCTTATTGGTCCACAGGCTTACTC<br>GTCATGGTTTTACATTTTTCCAAAAGGTATTCGACACTTTTTGAACTATACCAAA<br>GATACATATAATGATCCAGTCATTTACGTTACTGAGAATGGGGTTGACAACTACA<br>ATAATGAATCTCAACCAATTGAAGAGGCACTTCAAGATGATTTCAGGATTTCGTA<br>CTATAAAAAGCATATGTGGAATGCACTAGGATCTCTCAAGAACTACGGTGTTAAA<br>CTCAAAGGTTATTTTGCATGGTCATATTTAGACAACTTCGAATGGAATATTGGTT<br>ATACATCAAGATTTGGGTTGTACTATGTAGACTACAAAAATAACCTAACAAGGTA<br>TCCCAAGAAATCGGCTCATTGGTTCACAAAATTCCTGAATATATCGGTTAATGCA<br>AATAATATCTATGAGCTTACATCAAAGGATTCAAGGAAGGTTGGCAAATTCTATG<br>TGATGTACCCATACGATGTTCCAGATTACGCTGGATCTGGCGAAGGCAGAGGATC<br>TCTGCTGACATGCGGCGACGTGGAAGAGAATCCTGGACCTGGAAGCGGAGGAATG<br>GTGAGCAAGGGCGAGGCAGTGATCAAGGAGTTCATGCGGTTCAAGGTGCACATGG<br>AGGGCTCCATGAACGGCCACGAGTTCGAGATCGAGGGCGAGGGCGAGGGCCGCCC<br>CTACGAGGGCACCCAGACCGCCAAGCTGAAGGTGACCAAGGGTGGCCCCCTGCCC<br>TTCTCCTGGGACATCCTGTCCCCTCAGTTCATGTACGGCTCCAGGGCCTTCACCA<br>AGCACCCCGCCGACATCCCCGACTACTATAAGCAGTCCTTCCCCGAGGGCTTCAA<br>GTGGGAGCGCGTGATGAACTTCGAGGACGGCGGCGCCGTGACCGTGACCCAGGAC<br>ACCTCCCTGGAGGACGGCACCCTGATCTACAAGGTGAAGCTCCGCGGCACCAACT<br>TCCCTCCTGACGGCCCCGTAATGCAGAAGAAGACAATGGGCTGGGAAGCGTCCAC<br>CGAGCGGTTGTACCCCGAGGACGGCGTGCTGAAGGGCGACATTAAGATGGCCCTG

	CGCCTGAAGGACGGCGGCCGCTACCTGGCGGACTTCAAGACCACCTACAAGGCCA<br>AGAAGCCCGTGCAGATGCCCGGCGCCTACAACGTCGACCGCAAGTTGGACATCAC<br>CTCCCACAACGAGGACTACACCGTGGTGGAACAGTACGAACGCTCCGAGGGCCGC<br>CACTCCACCGGCGGCATGGACGAGCTGTACAAGTCCGGACTCAGATCTCGAGCTC<br>AAGCTTCGAATTCTGCAGTCGACGGTACCGCGGGCCCGGGATCCACCGGATCTAG<br>ATAAGCTAGCAATAAAGGATCGTTTATTTTCATTGGAAGCGTGTGTTGGTTTTTT<br>GATCACTCGAGGCGGCCGCAAAAAAGTAACGACGCGACGACGTAACTCTTGATTA<br>CGTGGTCGCGTCGTTACGGTGTTTCGTCCTTTCCACAAGATATATAAAGCCAAGA<br>AATCGAAATACTTTCAAGTTACGGTAAGCATATGATAGTCCATTTTAAAACATAA<br>TTTTAAAACTGCAAACTACCCAAGAAATTATTACTTTCTACGTCACGTATTTTGT<br>ACTAATATCTTTGTGTTTACAGTCAAATTAATTCTAATTATCTCTCTAACAGCCT<br>TGTATCGTATATGCAAATATGAAGGAATCATGGGAAATAGGCCCTCTTCCTGCCC<br>GACCTTGGCGCGCGCTCGGCGCGCGGTCACGCTCCGTCACGTGGTGCGTTTTGCC<br>TGCGCGTCTTTCCACTGGGGCCGGGGCGGCGCGGAGGGTTCAGACGCGGGCTCTT<br>ACTCAAACGCTGATCCCCGAAACTACCGAGCAGTGTCTGCGCGACGCGAAAGCAT<br>TTTTGGAGCGAGGGGCCATGGTGCCCATGGGATTCCCACGGGTGTTGGACTCGGG<br>CGCGCCGATATCAGATCTGGGAAACCAGATGATGGAGGTACCCACTCCCTCTATG<br>CGCGCTCGCTCACTCACTCGGCCCTGCCGGCCAGAGGCCGGCAGTCTGGAGACCT<br>TTGGTCTCCAGGGCCGAGTGAGTGAGCGAGCGCGCATAGAGGGAGTGGGTAGGAC<br>GCG

Plasmid	Nucleic acid sequence (5’ → 3’)
**pHJW410**<br><br>T7 promoter<br>SpyCatcher001<br>Δ<br>DARPin E01<br>His6<br><br><br>Backbone:<br>pRSET	CCCGCGAAATTAATACGACTCACTATAGGGAGACCACAACGGTTTCCCTCTAGAA<br>ATAATTTTGTTTAACTTTAAGAAGGAGATATACATATGATGAGCGGTGATAGTGC<br>TACCCATATTAAATTCTCAAAACGTGATGAGGACGGCAAAGAGTTAGCTGGTGCA<br>ACTATGGAGTTGCGTGATTCATCTGGTAAAACTATTAGTACATGGATTTCAGATG<br>GACAAGTGAAAGATTTCTACCTGTATCCAGGAAAATATACATTTGTCGAAACCGC<br>AGCACCAGACGGTTATGAGGTAGCAACTGCTATTACCTTTACAGTTAATGAGCAA<br>GGTCAGGTTACTGTAAATGGCGGCAGCGGTGGCGCTAGCGCCGGCGACCTGGGCA<br>AGAAGCTGCTGGAAGCCGCCAGAGCCGGACAGGACGACGAAGTGCGGATCCTGAT<br>GGCCAACGGCGCCGACGTGAACGCCGACGATACCTGGGGCTGGACCCCTCTGCAC<br>CTGGCCGCCTATCAGGGCCACCTGGAAATCGTGGAAGTGCTGCTGAAGAACGGGG<br>CCGATGTGAATGCCTACGACTACATCGGCTGGACACCCCTGCATCTCGCTGCCGA<br>CGGCCACCTCGAGATTGTCGAGGTCCTGCTGAAAAATGGCGCTGATGTGAACGCC<br>AGCGATTATATCGGCGACACCCCCCTCCATCTGGCCGCCCACAACGGCCATCTGG<br>AAATTGTCGAAGTCCTCCTCAAGCACGGCGCAGATGTCAACGCCCAGGACAAGTT<br>CGGCAAGACCGCCTTCGACATCAGCATCGACAACGGCAACGAGGACCTGGCCGAG<br>ATCCTCCAGCATCATCACCATCACCATTAAGCGGCCG

Plasmid	Nucleic acid sequence (5’ → 3’)
**pHJW415**<br><br>T7 promoter<br>SpyCatcher001<br>Δ<br>DARPin Ec1<br>His6<br><br><br>Backbone:<br>pRSET	CCCGCGAAATTAATACGACTCACTATAGGGAGACCACAACGGTTTCCCTCTAGAA<br>ATAATTTTGTTTAACTTTAAGAAGGAGATATACATATGATGAGCGGTGATAGTGC<br>TACCCATATTAAATTCTCAAAACGTGATGAGGACGGCAAAGAGTTAGCTGGTGCA<br>ACTATGGAGTTGCGTGATTCATCTGGTAAAACTATTAGTACATGGATTTCAGATG<br>GACAAGTGAAAGATTTCTACCTGTATCCAGGAAAATATACATTTGTCGAAACCGC<br>AGCACCAGACGGTTATGAGGTAGCAACTGCTATTACCTTTACAGTTAATGAGCAA<br>GGTCAGGTTACTGTAAATGGCGGCAGCGGTGGCGCTAGCGATCTGGGTAAAAAAC<br>TGCTGGAAGCAGCACGTGCAGGTCAGGATGATGAAGTTCGTATTCTGGTTGCAAA<br>TGGTGCAGATGTGAATGCCTATTTTGGCACCACACCGCTGCATCTGGCAGCAGCA<br>CATGGTCGTCTGGAAATTGTTGAAGTTCTGCTGAAAAACGGTGCCGATGTTAATG<br>CACAGGATGTTTGGGGTATTACCCCGTTACACCTGGCAGCATATAACGGTCATTT<br>AGAAATCGTGGAAGTGCTGCTTAAATATGGCGCAGATGTTAACGCCCATGATACC<br>CGTGGTTGGACCCCTCTGCATTTAGCAGCAATTAATGGTCACCTGGAAATCGTAG<br>AGGTGCTTCTGAAAAATGTGGCCGATGTGAACGCGCAGGATCGTAGCGGTAAAAC<br>CCCGTTTGATCTGGCAATTGATAATGGCAATGAAGATATTGCAGAGGTGCTGCAG<br>AAAGCAGCAAAACTGAATCATCATCACCATCACCATTAAGTAAGCG

Plasmid	Nucleic acid sequence (5’ → 3’)
**pHJW416**<br><br>T7 promoter<br>SpyCatcher001<br>Δ<br>DARPin 9.29<br>His6<br><br><br>Backbone:<br>pRSET	CCCGCGAAATTAATACGACTCACTATAGGGAGACCACAACGGTTTCCCTCTAGAA<br>ATAATTTTGTTTAACTTTAAGAAGGAGATATACATATGATGAGCGGTGATAGTGC<br>TACCCATATTAAATTCTCAAAACGTGATGAGGACGGCAAAGAGTTAGCTGGTGCA<br>ACTATGGAGTTGCGTGATTCATCTGGTAAAACTATTAGTACATGGATTTCAGATG<br>GACAAGTGAAAGATTTCTACCTGTATCCAGGAAAATATACATTTGTCGAAACCGC<br>AGCACCAGACGGTTATGAGGTAGCAACTGCTATTACCTTTACAGTTAATGAGCAA<br>GGTCAGGTTACTGTAAATGGCGGCAGCGGTGGCGCTAGCGATCTGGGTAAAAAAC<br>TGCTGGAAGCAGCACGTGCAGGTCAGGATGATGAAGTTCGTATTCTGATGGCAAA<br>TGGTGCAGATGTTAATGCCCATGATTTTTATGGTATTACACCGCTGCATCTGGCA<br>GCAAATTTTGGTCATCTGGAAATTGTTGAAGTGCTGCTGAAACATGGTGCCGATG<br>TGAACGCATTTGATTATGATAATACCCCGTTACACCTGGCAGCAGATGCAGGCCA<br>TTTAGAAATCGTGGAAGTGTTACTGAAATATGGCGCAGACGTGAATGCAAGCGAT<br>CGTGATGGTCATACCCCTCTGCATTTAGCAGCCCGTGAAGGCCACCTGGAAATAG<br>TAGAGGTTCTGCTTAAAAACGGTGCGGATGTAAATGCGCAGGATAAATTTGGTAA<br>AACCGCCTTTGATATCAGCATCGATAATGGCAATGAAGATCTGGCCGAAATCCTG<br>CAGAAACTGAATCACATCATCACCATCACCATTAAGTAAGCG

Plasmid	Nucleic acid sequence (5’ → 3’)
**pHJW417**<br><br>T7 promoter<br>SpyCatcher001<br>Δ<br>DARPin D55.2<br>His6<br><br><br>Backbone:<br>pRSET	CCCGCGAAATTAATACGACTCACTATAGGGAGACCACAACGGTTTCCCTCTAGAA<br>ATAATTTTGTTTAACTTTAAGAAGGAGATATACATATGATGAGCGGTGATAGTGC<br>TACCCATATTAAATTCTCAAAACGTGATGAGGACGGCAAAGAGTTAGCTGGTGCA<br>ACTATGGAGTTGCGTGATTCATCTGGTAAAACTATTAGTACATGGATTTCAGATG<br>GACAAGTGAAAGATTTCTACCTGTATCCAGGAAAATATACATTTGTCGAAACCGC<br>AGCACCAGACGGTTATGAGGTAGCAACTGCTATTACCTTTACAGTTAATGAGCAA<br>GGTCAGGTTACTGTAAATGGCGGCAGCGGTGGCGCTAGCGATCTGGGTAAAAAC<br>TGCTGGAAGCAGCACGTGCAGGTCAGGATGATGAAGTTCGTATTCTGATGGCAAA<br>TGGTGCAGATGTTAATGCCATGGATATTCTGGGTCTGACACCGCTGCATCTGGCA<br>GCAGTTGCCGGTCATCTGGAAATTGTTGAAGTTCTGCTGAAAAATAGCGCTGATG<br>TGAATGCGATTGATGAAGATGGCGAAACCCCTCTGCATCTGAGCGCAGCAATGGG<br>CCATCTGAAAATCGTGGAAGTGCTGTTAAAACATGGTGCCGATGTGAACGCACAG<br>GATAAATTTGGTAAAACCGCCTTTGATATCAGCATCGATTATGGCAATGAAGATC<br>TGGCAGAAATCCTGCAGAAACTGAATCATCATCACCATCACCATTAAGTAAGCG

Plasmid	Nucleic acid sequence (5’ → 3’)
**pHJW422**<br><br>T7 promoter<br>SpyCatcher003<br>DARPin E01<br>His6<br><br><br>Backbone:<br>pRSET	CCCGCGAAATTAATACGACTCACTATAGGGAGACCACAACGGTTTCCCTCTAGAA<br>ATAATTTTGTTTAACTTTAAGAAGGAGATATACATATGATGGTGACCACACTGTC<br>CGGACTTTCTGGTGAGCAGGGACCATCCGGCGACATGACCACAGAAGAGGATTCT<br>GCCACACACATCAAGTTCAGCAAACGCGACGAAGACGGTAGAGAGTTGGCAGGGG<br>CAACCATGGAACTGCGCGATAGCTCCGGCAAAACCATAAGCACATGGATCTCCGA<br>CGGCCATGTTAAGGATTTCTACCTGTATCCCGGCAAGTACACCTTTGTGGAGACA<br>GCAGCACCAGACGGATATGAGGTTGCAACCCCTATAGAATTTACAGTGAACGAGG<br>ACGGACAGGTTACCGTGGATGGAGAGGCAACAGAAGGCGATGCACACACCGGCAG<br>CGGTGGCGCTAGCGCCGGCGACCTGGGCAAGAAGCTGCTGGAAGCCGCCAGAGCC<br>GGACAGGACGACGAAGTGCGGATCCTGATGGCCAACGGCGCCGACGTGAACGCCG<br>ACGATACCTGGGGCTGGACCCCTCTGCACCTGGCCGCCTATCAGGGCCACCTGGA<br>AATCGTGGAAGTGCTGCTGAAGAACGGGGCCGATGTGAATGCCTACGACTACATC<br>GGCTGGACACCCCTGCATCTCGCTGCCGACGGCCACCTCGAGATTGTCGAGGTCC<br>TGCTGAAAAATGGCGCTGATGTGAACGCCAGCGATTATATCGGCGACACCCCCCT<br>CCATCTGGCCGCCCACAACGGCCATCTGGAAATTGTCGAAGTCCTCCTCAAGCAC<br>GGCGCAGATGTCAACGCCCAGGACAAGTTCGGCAAGACCGCCTTCGACATCAGCA<br>TCGACAACGGCAACGAGGACCTGGCCGAGATCCTCCAGCATCATCACCATCACCA<br>TTAAGCGGCCG

Plasmid	Nucleic acid sequence (5' → 3')
**pHJW423**<br><br>T7 promoter<br>SpyCatcher001<br>DARPin E01<br>His6<br><br>Backbone:<br>pRSET	CCCGCGAAATTAATACGACTCACTATAGGGAGACCACAACGGTTTCCCTCTAGAA<br>ATAATTTTGTTTAACTTTAAGAAGGAGATATACATATGATGGCCGGCGTTGATAC<br>CTTATCAGGTTTATCAAGTGAGCAAGGTCAGTCCGGTGATATGACAATTGAAGAA<br>GATAGTGCTACCCATATTAAATTCTCAAAACGTGATGAGGACGGCAAAGAGTTAG<br>CTGGTGCAACTATGGAGTTGCGTGATTCATCTGGTAAAACTATTAGTACATGGAT<br>TTCAGATGGACAAGTGAAAGATTTCTACCTGTATCCAGGAAAATATACATTTGTC<br>GAAACCGCAGCACCAGACGGTTATGAGGTAGCAACTGCTATTACCTTTACAGTTA<br>ATGAGCAAGGTCAGGTTACTGTAAATGGCAAAGCAACTAAAGGTGACGCTCATAT<br>TACCGGTGGCAGCGGTGGCGCTAGCGCCGGCGACCTGGGCAAGAAGCTGCTGGAA<br>GCCGCCAGAGCCGGACAGGACGACGAAGTGCGGATCCTGATGGCCAACGGCGCCG<br>ACGTGAACGCCGACGATACCTGGGGCTGGACCCCTCTGCACCTGGCCGCCTATCA<br>GGGCCACCTGGAAATCGTGGAAGTGCTGCTGAAGAACGGGGCCGATGTGAATGCC<br>TACGACTACATCGGCTGGACACCCCTGCATCTCGCTGCCGACGGCCACCTCGAGA<br>TTGTCGAGGTCCTGCTGAAAAATGGCGCTGATGTGAACGCCAGCGATTATATCGG<br>CGACACCCCCCTCCATCTGGCCGCCCACAACGGCCATCTGGAAATTGTCGAAGTC<br>CTCCTCAAGCACGGCGCAGATGTCAACGCCCAGGACAAGTTCGGCAAGACCGCCT<br>TCGACATCAGCATCGACAACGGCAACGAGGACCTGGCCGAGATCCTCCAGCATCA<br>TCACCATCACCATTAAGCGGCCG

Plasmid	Nucleic acid sequence (5' → 3')
**pHJW424**<br><br>T7 promoter<br>SpyCatcher003<br>Δ<br>DARPin E01<br>His6<br><br>Backbone:<br>pRSET	CCCGCGAAATTAATACGACTCACTATAGGGAGACCACAACGGTTTCCCTCTAGAA<br>ATAATTTTGTTTAACTTTAAGAAGGAGATATACATATGATGAGCGGTGATTCTGC<br>CACACACATCAAGTTCAGCAAACGCGACGAAGACGGTAGAGAGTTGGCAGGGGCA<br>ACCATGGAACTGCGCGATAGCTCCGGCAAAACCATAAGCACATGGATCTCCGACG<br>GCCATGTTAAGGATTTCTACCTGTATCCCGGCAAGTACACCTTTGTGGAGACAGC<br>AGCACCAGACGGATATGAGGTTGCAACCCCTATAGAATTTACAGTGAACGAGGAC<br>GGACAGGTTACCGTGGATGGAGGCAGCGGTGGCGCTAGCGCCGGCGACCTGGGCA<br>AGAAGCTGCTGGAAGCCGCCAGAGCCGGACAGGACGACGAAGTGCGGATCCTGAT<br>GGCCAACGGCGCCGACGTGAACGCCGACGATACCTGGGGCTGGACCCCTCTGCAC<br>CTGGCCGCCTATCAGGGCCACCTGGAAATCGTGGAAGTGCTGCTGAAGAACGGGG<br>CCGATGTGAATGCCTACGACTACATCGGCTGGACACCCCTGCATCTCGCTGCCGA<br>CGGCCACCTCGAGATTGTCGAGGTCCTGCTGAAAAATGGCGCTGATGTGAACGCC<br>AGCGATTATATCGGCGACACCCCCCTCCATCTGGCCGCCCACAACGGCCATCTGG<br>AAATTGTCGAAGTCCTCCTCAAGCACGGCGCAGATGTCAACGCCCAGGACAAGTT<br>CGGCAAGACCGCCTTCGACATCAGCATCGACAACGGCAACGAGGACCTGGCCGAG<br>ATCCTCCAGCATCATCACCATCACCATTAAGCGGCCG

References

(1.) Gomez, E. J., Gerhardt, K., Judd, J., Tabor, J. J., and Suh, J. (**2016**) Light-Activated Nuclear Translocation of Adeno-Associated Virus Nanoparticles Using Phytochrome B for Enhanced, Tunable, and Spatially Programmable Gene Delivery, *ACS Nano 10*, 225-237.

(2.) Link, N., Aubel, C., Kelm, J. M., Marty, R. R., Greber, D., Djonov, V., Bourhis, J., Weber, W., and Fussenegger, M. (**2006**) Therapeutic protein transduction of mammalian cells and mice by nucleic acid-free lentiviral nanoparticles, *Nucleic Acids Research 34*, e16-e16.

(3.) Hörner, M., Jerez-Longres, C., Hudek, A., Hook, S., Yousefi, O. S., Schamel, W. W. A., Hörner, C., Zurbriggen, M. D., Ye, H., Wagner, H. J., and Weber, W. (**2021**) Spatiotemporally confined red light-controlled gene delivery at single-cell resolution using adeno-associated viral vectors, *Science Advances 7*, eabf0797.
